# A NR2E1‐interacting peptide of LSD1 inhibits the proliferation of brain tumour initiating cells

**DOI:** 10.1111/cpr.13350

**Published:** 2022-11-02

**Authors:** Rong Hu, Umar Farook Shahul Hameed, Xiang Sun, Balakrishnan Shenbaga Moorthy, Wen Zhang, Philip D. Jeffrey, Li Zhou, Xin Ma, Fangjin Chen, Jianfeng Pei, Pankaj K. Giri, Yonggao Mou, Kunchithapadam Swaminathan, Ping Yuan

**Affiliations:** ^1^ Guangdong Institute of Gastroenterology Guangzhou People's Republic of China; ^2^ Guangdong Provincial Key Laboratory of Colorectal and Pelvic Floor Disease, The Sixth Affiliated Hospital Sun Yat‐sen University Guangzhou People's Republic of China; ^3^ Li Ka Shing Institute of Health Sciences The Chinese University of Hong Kong Hong Kong SAR China; ^4^ Department of Biological Sciences National University of Singapore Singapore Singapore; ^5^ Department of Medical Informatics, Zhongshan School of Medicine Sun Yat‐sen University Guangzhou China; ^6^ Center for Stem Cell Biology and Tissue Engineering, Key Laboratory for Stem Cells and Tissue Engineering, Ministry of Education Sun Yat‐sen University Guangzhou China; ^7^ Department of Molecular Biology Princeton University Princeton New Jersey USA; ^8^ Department of Chemical Pathology The Chinese University of Hong Kong Hong Kong SAR China; ^9^ Center for Quantitative Biology, Academy for Advanced Interdisciplinary Studies Peking University Beijing People's Republic of China; ^10^ Faculty of Life Sciences and Biotechnology South Asian University New Delhi India; ^11^ Department of Neurosurgery/Neuro‐oncology Sun Yat‐sen University Cancer Center, State Key Laboratory of Oncology in South China, Collaborative Innovation Center for Cancer Medicine Guangzhou China

## Abstract

**Objectives:**

Elimination of brain tumour initiating cells (BTICs) is important for the good prognosis of malignant brain tumour treatment. To develop a novel strategy targeting BTICs, we studied NR2E1(TLX) involved self‐renewal mechanism of BTICs and explored the intervention means.

**Materials and Methods:**

NR2E1 and its interacting protein‐LSD1 in BTICs were studied by gene interference combined with cell growth, tumour sphere formation, co‐immunoprecipitation and chromatin immunoprecipitation assays. NR2E1 interacting peptide of LSD1 was identified by Amide Hydrogen/Deuterium Exchange and Mass Spectrometry (HDX‐MS) and analysed by *in vitro* functional assays. The *in vivo* function of the peptide was examined with intracranial mouse model by transplanting patient‐derived BTICs.

**Results:**

We found NR2E1 recruits LSD1, a lysine demethylase, to demethylate mono‐ and di‐methylated histone 3 Lys4 (H3K4me/me2) at the *Pten* promoter and repress its expression, thereby promoting BTIC proliferation. Using Amide Hydrogen/Deuterium Exchange and Mass Spectrometry (HDX‐MS) method, we identified four LSD1 peptides that may interact with NR2E1. One of the peptides, LSD1‐197‐211 that locates at the LSD1 SWIRM domain, strongly inhibited BTIC proliferation by promoting *Pten* expression through interfering NR2E1 and LSD1 function. Furthermore, overexpression of this peptide in human BTICs can inhibit intracranial tumour formation.

**Conclusion:**

Peptide LSD1‐197‐211 can repress BTICs by interfering the synergistic function of NR2E1 and LSD1 and may be a promising lead peptide for brain tumour therapy in future.

## Graphical abstract

- NR2E1 and LSD1 complex directly represses *Pten* in BTICs by demethylating H3K4me and H3K4me2 at its promoter, and thus promotes the proliferation of BTICs.
- LSD1‐197‐211 peptide interferes the interaction between NR2E1 and LSD1 and restores Pten expression, which in turn inhibits the proliferation of BTICs.

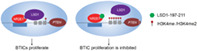

## 1 INTRODUCTION

Malignant brain cancers, like glioblastoma (GBM), are highly heterogeneous and aggressive. They are resistant to chemotherapy and radiation therapy and show a high chance of relapse. The patient survival time is only about 15 months after diagnosis. This situation has lasted over the past decades although multiple novel therapeutic means have been employed.^1^, ^2^ It has been suggested that cancer initiating cells (CICs) with stem cell properties underlie the heterogeneity of malignant tumours.^3^ They are less differentiated and resistant to chemotherapy and radiation treatment. They are thought to be the “root” of tumour occurrence and are responsible for the growth and relapse of tumours. Targeting CICs to treat cancer may help to improve the outcome of clinical therapies.^4^

Researches have revealed that the recurrence of high‐grade gliomas is due to the existence of brain tumour initiating cells (BTICs). BTICs were among the first CICs derived from a solid tumour.^5^, ^6^ BTICs express the neural stem cell surface marker CD133. And as little as 100 BTICs could initiate phenocopies of the original tumours in a NOD.CB17‐Prkdcscid/J (NOD SCID) mouse brain.^6^ BTICs and neural stem cells (NSCs) share several similarities, and it has been suggested that BTICs hijack the self‐renewal mechanisms of NSCs to support their proliferation.^7^ Many studies have shown that the factors important for NSC maintenance also play important roles in brain tumorigenesis. For example, Nestin, which labels NSCs in adult mouse brain, also marks BTICs in glioblastoma and is required for the long‐term sustenance of tumour growth.^8^

NR2E1(TLX), an orphan nuclear receptor, is essential for the self‐renewal of BTICs. NR2E1‐positive glioma cells can initiate brain tumours and form spheres in suspension culture.^3^ Depletion of *Nr2e1* in mouse primary tumours significantly extended animal survival time. Interestingly, GBM patients express a high level of NR2E1 which is correlated with poor survival time.^3^ NR2E1 may therefore be a valuable target for brain tumour therapy. Just like Nestin, NR2E1 is highly expressed at the hippocampal dentate gyrus and the subventricular zone. It is required for the maintenance and self‐renewal of neural stem cells.^9^ In NSCs, NR2E1 interacts with LSD1, a histone demethylase, and recruits it to the promoter of *Pten*. LSD1 then demethylates mono‐ and di‐methylated histone 3 Lys 4 (H3K4) and removes these active epigenetic markers from the regions to silence the expression of *Pten*, a gene that induces apoptosis, regulates the cell cycle and functions as a tumour repressor. Through coordinated repression of *Pten*, NR2E1 and LSD1 contribute to the proliferation of NSCs and retinoblastoma cells.^9^, ^10^ *Pten* is an important mis‐regulated tumour suppressor gene in almost all types of cancers. It is now an open and interesting question whether a similar mechanism is also employed in BTICs.

Lysine‐specific histone demethylase LSD1 (also named AOF2 or KDM1A or BHC110) is a FAD dependent lysine demethylase. LSD1 can demethylate mono‐ and di‐methylated H3K4 in a complex with CoREST, but shifts its targets to mono‐ and di‐methylated H3K9 when it partners with the androgen receptor (AR).^11^, ^12^, ^13^, ^14^ Thus, by changing partners, LSD1 is involved in both gene activation and gene repression. The N‐terminus of LSD1 is a non‐structural element and contains a putative nuclear localization signal. Following this region is the Swip3p/Rsc8p/Moira (SWIRM) domain. After the SWIRM domain is an oxidase domain which is involved in demethylating proteins. LSD1 is also linked to the growth of glioblastoma and its inhibition increases the sensitivity of glioblastoma cells to histone deacetylase (HDAC) inhibitor treatment.^15^ Since NR2E1 and LSD1 both play important roles in glioblastoma, we set out to investigate whether BTICs employ the same NR2E1‐LSD1 mechanism, as in NSCs, to regulate BTIC proliferation.

## 2 MATERIALS AND METHODS

### 2.1 Cell culture

Brain tumour initiating cells (BTICs) derived from Nestin‐TV‐a mice were received as a gift from Dr. Haikun Liu's lab.^3^ To culture BTICs in monolayer, the cell culture plate was coated with laminin and poly‐L‐lysine. Cells were grown in DMEM/F12 medium plus 20 ng/ml epidermal growth factor (EGF), 10 ng/ml fibroblast growth factor (FGF‐2), B27 and insulin‐transferrin‐selenium supplements (ITSS). Cells were digested with accutase for passage. To culture brain tumour initiating cells in sphere, cells were seeded in a low attached cell culture plate (Corning) in the above medium without any coating. Human BTICs were derived from surgical samples of patients who were diagnosed with IV glioblastoma with the approval of the Sun Yat‐sen University Cancer Center Ethics Committee in accordance with ICH‐GCP principle and related Chinese regulation. The tumour tissue was minced and digested by 0.25% Trypsin–EDTA (GIBCO). The digested sample was passed through 40 μm strainer to collect single cells. The cells were washed by PBS and then grew in mouse BTIC culture medium in suspension and tumour spheres formed 1 day later. The tumour spheres were collected and digested by StemPro Accutase (GIBCO) and then passaged at 1:3 ratio with fresh medium. To induce BTIC differentiation, the culture medium was changed to DMEM/F12 medium plus 0.5% FBS and 1 μM all‐trans retinoic acid (Sigma, R2625). Three days later, the cells were harvested for immunostaining of differentiated cell marks.

### 2.2 Cloning

shRNA constructs were generated as previously described.^16^ The sequences are as follows: *Nr2e1* shRNA1 GGCTGTATCTGGCATGAAT, shRNA2 CGTGGACACAAGGAAGACAAT; *Lsd1* shRNA1 CACAAGGAAAGCTAGAAGA, shRNA2 CCACAAGTCAAACCTTTATTT; *Pten* shRNA (TRCN0000322421, Sigma) CGACTTAGACTTGACCTATAT and control shRNA GATGAAATGGGTAAGTACA. The shRNAs were cloned into the pSuper‐puro plasmid (Addgene) between the BglII and HindIII sites or lentiviral vector between Age1 and EcoRI sites. For RNAi rescue assay, we used Mut Express II Fast Mutagenesis Kit V2 (C214, Vazyme) to generate shRNA immune cDNA and then cloned the cDNA into G418 resistant pCDH lentiviral vector. The mutations were verified by sequencing. For transient gene overexpressing clones, *NR2E1*, *LSD1* and LSD1 peptide cDNAs were cloned into the pCAG‐puromycin or pCAG‐hygromycin plasmids. LSD1 peptide deletion clones were generated in the pCAG‐puro vector by two‐step PCR. For lentivirus vector construction, LSD1‐197‐211 peptide cDNA was cloned into pLenti‐TRE‐EGFP‐EF1‐rtTA3‐IRES‐Puro plasmid at EcoRI restriction sites. The clone was sequenced and confirmed by forward primer ACGGTGGGAGGCCTATATAAGC and reverse primer CGTCGCCGTCCAGCTCGACCAG.

### 2.3 Reverse transcription and real‐time PCR

Reverse transcription was performed with 2 μg of total RNA using the PrimeScript RT reagent kit (Takara). Real‐time PCR analysis was performed by using the ABI Prism 7900HT machine (Applied Biosystems) with the SYBR Green mixture (Takara). For each primer, only one correct size band was formed. All experiments were repeated three times independently. The final results were normalized against the expression of *β‐Actin* or *Gapdh*. Student's t‐test was used for the statistical significance appraisal.

### 2.4 Cell growth assay

10^6^ BTICs were transfected with 2 μg plasmids of interests by electroporation with Amaxa cell line Nucleofector Kit V using nucleofector II. The transfected cells were seeded on a poly‐l‐lysine and laminin treated 6‐well plate. After 24 h, the transfected cells were selected with 1 μg/ml puromycin. After 3 days, the floating dead cells were washed away and the viability of cells was quantitated using MTT assay or CCK‐8 assay by following manufacturer's protocol. All experiments were repeated three times independently. Student's *t*‐test was used for the statistical significance appraisal.

### 2.5 BrdU incorporation assay

BTICs were cultured with a 5‐bromo‐2′‐deoxyuridine (BrdU)‐labeling reagent (Meilunbio, MB3126‐1) and stained with an anti‐BrdU antibody (Proteintech, 66241‐1‐Ig) as first antibody and Alexa Fluor 488 goat anti‐mouse (A11001, Invitrogen) as the secondary antibody according to the manufacturer's instructions. The images were captured by OLYMPUS IX73 fluorescence microscopy.

### 2.6 Chromatin immunoprecipitation

ChIP assay was carried out as described previously with slight modification.^17^ Briefly, cells were fixed with 1% (w/v) formaldehyde for 10 min at room temperature, and 125 mM glycine was used to inactivate formaldehyde. Chromatins were sonicated to generate average fragment sizes from 200 to 600 bp and immunoprecipitated using the anti‐NR2E1 (a gift from Liu's lab), anti‐LSD1 (ab17721, Abcam), anti‐H3K4me (ab8895, Abcam), anti‐H3K4me2 (ab7766, Abcam) antibodies and control IgG or control GFP. The ChIP enriched DNA and input were then decrosslinked and proteins were digested by proteinase K. DNAs were purified by phenol: chloroform extractions and followed by ChIP‐qPCR analysis using the ABI PRISM 7900 sequence detection system and Kappa SYBR green master mix (Takara). The values of each real‐time PCR assay were normalized with its own input value and then compared with the IgG or GFP value to get the enrichment fold. PCR primers were designed to amplify the promoter regions of mouse *Pten* and control according to previous research.^18^ Each experiment was performed three times independently. Student's *t*‐test was used for the statistical significance appraisal.

### 2.7 Western blot

Western blot was performed by following standard protocols.^19^ Total protein was collected by lysing cells with RIPA buffer containing 0.2 M NaCl, 1% SDS, 1 mM PMSF and 0.1 M DTT. Proteins were then separated by SDS‐PAGE and transferred to PVDF membrane (Pall). The membrane was blocked with TBS + 0.1% Tween with 5% non‐fat milk (BD) and then blotted with proper primary antibody in TBS + 0.1% Tween at cold room overnight. The primary antibodies and dilutions were: rabbit anti‐LSD1 (ab17721, Abcam) at 1/1000, rabbit anti‐NR2E1 (a gift from Haikun Liu's lab) at 1/500, rabbit anti‐PTEN (ab31392, Abcam) at 1/1000, mouse anti‐p21 (CS2948, CST) at 1/1000, mouse anti‐ACTIN (sc‐47778, Santa Cruz) at 1/1000, mouse anti‐GAPDH (sc‐137179, Santa Cruz) at 1/1000. Suitable secondary antibodies, such as anti‐rabbit or anti‐mouse antibodies conjugated with HRP, were used for ECL detection (Amershan).

### 2.8 Immunofluorescence staining

The cells were fixed with 4% paraformaldehyde (PFA) for 30 min at 4°C, and then washed in cold PBS for 5 min 3 times. The nuclei were subsequently permeabilized with PBS with 0.5% Triton X‐100 for 30 min. Next, the cells were blocked with 1% BSA in PBS for 1 h. The cells were incubated with primary antibody overnight at 4°C. Primary antibodies used were cleaved CASPASE 3 antibody (9664S, CST), Ki67 antibody (ab15580, Abcam), NESTIN antibody (Santa Cruz, sc‐58813), NR2E1 antibody (Santa Cruz, sc‐377240X), GFAP antibody (Millipore, 5541) and TUJ‐1 (abcam, ab1445). After wash, the cells were incubated with anti‐rabbit secondary antibody conjugated with proper Alexa Fluor label for 1 h at room temperature in darkness. The nuclei were counterstained with DAPI. The cells were imaged with Olympus IX‐73 immunofluorescence microscope.

### 2.9 Co‐immunoprecipitation

Immunoprecipitation assays were performed with whole‐cell lysates from BTICs or targeted cells transfected with overexpression plasmids. Anti‐NR2E1 (ab30942, Abcam), anti‐LSD1 (ab17721, Abcam), anti‐FLAG (#F1804, SIGMA) and anti‐HA (ab18181, Abcam) antibodies were used to pull down protein complexes. Immunoprecipitated complexes, bound to the corresponding antibody, were washed extensively with 0.1% Triton X‐100 buffer (50 mM Tris–HCl at pH 8, 150 mM NaCl, 1 mM EDTA, 0.1% Triton X‐100, 10% glycerol plus Roche protease inhibitor cocktail). The interacting protein bands were resolved with 10% SDS‐PAGE gel and transferred to the PVDF membrane, followed by detection with an appropriate primary antibody, an HRP‐conjugated second antibody, and an ECL detection reagent.

### 2.10 In vitro limiting dilution assay

Doxycycline inducible control GFP or LSD1‐197‐211 lentivirus transduced BTICs were seeded at density of 5, 50, 100, 250 or 500 cells per well into a low attached 24‐well plate. 5 μg/ml Doxycycline was added into the plate on every other day. The number of spheres were counted at day 7 after seeding. Stem cell frequency was analysed by using extreme limiting dilution analysis as described^20^, ^21^ with software ELDA (http://bioinf.wehi.edu.au/software/elda). Each experiment was performed three times independently. Student's *t*‐test was used for the statistical significance appraisal.

### 2.11 Animals and intracranial transplantation

The animal experiments were performed with the approval of Animal Experimentation Ethics Committee (AEEC) in the Sixth Affiliated Hospital of Sun Yat‐sen University. 10^5^ doxycycline inducible GFP or LSD1‐197‐211‐GFP transduced human BTICs were intracranially transplanted into the frontal lobe of 6 to 8‐week‐old female nude mice (Beijing HFK Bioscience Co. Ltd, China). The cells were suspended in 5 μl PBS and injected into the right frontal lobe at 2 mm lateral and 1 mm anterior to bregma with 2.5 mm depth from the skull base. The mice were fed with water with or without fresh 2 mg/ml Doxycycline (DOX) daily from the second day after transplantation. The brain tumour growth was monitored by IVIS Spectrum imaging (PerkinElmer) after transplantation. The mouse brain was harvested after sacrificed by CO_2_ suffocation for hematoxylin–eosin staining. Mouse tumour volume was calculated as previously described by the formula *V* = *ab* ^2^/2, where a and b are the length and width of the tumour.^22^ The experiments have been performed twice independently with each group contained no less than five mice. Mouse survival curve was plotted by Graphpad prism 7.0 software.

### 2.12 Amide Hydrogen/Deuterium Exchange and Mass Spectrometry (HDX‐MS)

Human NR2E1 (GenScript) was cloned into pET22b to express protein with C‐terminal His tag. The protein was purified with Ni‐NTA beads and followed by gel filtration. Human LSD1 (ATCC) was cloned into pGEX6 to express protein with N‐terminal GST tag. The protein was purified with glutathione agarose and the GST tag was removed by precision protease digestion at 4°C overnight. The eluted protein was further purified by gel filtration. To study the peptide of LSD1 involved in the interaction with NR2E1, 50 μM of full length human LSD1 protein was incubated with 75 μM of human NR2E1 (residues 183–354) in buffer A (25 mM potassium phosphate and 5% glycerol at pH 7.5) for 12 h prior to the HDX experiments. 2 μl of LSD1 alone or in complex with NR2E1 was mixed with 18 μl of deuterated buffer A resulting in a final concentration of 90% D_2_O. Exchange reactions were carried out at 20°C for five different time points (0.5–10 min) and quenched by adding 40 μl of ice cold 0.1% trifluoro acetic acid to get a final pH of 2.5. 50 μl aliquot of the quenched samples containing 0.83 μM of LSD1 was injected into a chilled nano‐Ultra Performance Liquid Chromatography sample manager (test version, Waters), specially designed for HDX experiments. Online digestion was carried out using an immobilized pepsin column (Porozyme, ABI) in water containing 0.05% FA at a flow rate of 100 μl/min. The digested sample was desalted in a 2.1 × 5 mm C18 peptide microtrap (ACQUITY BEH C18 VanGuard Pre‐column, 1.7 μm, Waters) and eluted using a linear gradient of acetonitrile (8%–40%) in 0.1% FA, onto a reverse phase analytical column (ACQUITY UPLC BEH C18 Column, 1.0 × 100 mm, 1.7 μm, Waters) at 40 μl/min. Mass spectra were acquired over the m/z range 200–1700 and continuous instrument calibration was carried out using Glu‐Fibrinogen peptide (GFP) at 100 fmol/μl. Peptides were identified from MSE data of an undeuterated LSD1 sample using ProteinLynx Global Server (PLGS 2.4) (test version, Waters) and mapped onto subsequent deuteration experiments using prototype custom software (DynamX, Waters). The average number of deuterons exchanged for each of the pepsin digest fragments was obtained as described before.^23^ Exchange values were not corrected for the deuterium back‐exchange, that occurs during sample analysis and so all the results reported in this study are only from the relative deuterium level. The difference in deuterium uptake (subtracting the absolute deuterium level in LSD1 from the NR2E1:LSD1 samples) for 56 pepsin digest fragments at all the time points was plotted using Origin software (Origin Pro v.8.6, OriginLab). The percent difference in deuterium uptake for all of the pepsin digest fragments between LSD1 and NR2E1:LSD1 following 1 min HDX were shown below (Equation (1)) and mapped onto the crystal structure of LSD1 (PDB ID: 2Z3Y) using PyMOL (PyMOL Molecular Graphics System, Version 1.3, Schrodinger, LLC).

(1) $$\text{Percent difference}=\begin{array}{l} \frac{\text{deuterium uptake}\;\mathrm{by}\;\mathrm{LSD}1-\text{deuterium uptake}\;\mathrm{by}\;\mathrm{LSD}1:\mathrm{NR}2E1}{\text{total number of exchangeable amides}}\\ \times 100 \end{array}$$

### 2.13 Crystal structure determination

The SWIRM domain of human LSD1 residue 172–280, was subcloned into the pET15b vector between the EcoRI and BamH1 restriction sites, and the resulting plasmid was transformed into *E. coli* strain BL21. Cells were grown at 37°C in LB medium to an optical density of 0.6 at 600 nm and induced with 0.5 mM IPTG. The collected cells were lysed by French press in a buffer containing 50 mM Tris, pH 7.0, 150 mM NaCl, 1 mM EDTA and 1 mM dithiothreitol (DTT). After removing cell debris by centrifugation at 10,000 × g for 30 min, the supernatant was mixed with Ni‐NTA resin, and then poured into a column. After extensive wash, the SWIRM domain protein was released from the resin by 300 mM imidazole. Selenomethionine (SeMet) protein was expressed following the standard method.^24^ Native crystals were grown at 20°C by the hanging drop vapour diffusion method: 2 μl of protein at a concentration of 10 mg/ml was mixed with equal amount of reservoir buffer consisting of 0.1 M MgCl_2_, 0.1 M Tris pH 8.5, 25% (w/v) PEG 3350. SeMet derivative crystals were grown in reservoir buffer containing 0.8 M K/Na tartrate, 0.1 M MES pH 6, 2.5% (v/v) glycerol. Native SWIRM protein crystallizes in the space groups P2_1_2_1_2_1_ and P2_1_2_1_2 whereas SeMet SWIRM protein crystallized in space group I222. Diffraction data of native crystals and SeMet crystals were collected at beamline A1 and beamline F2 respectively in Cornell High Energy Synchrotron Source. All data were processed with DENZO/SCALEPACK. The structure was determined by multiwavelength anomalous dispersion (MAD) methods using SHELX and SHARP and refined using CNS.^25^ Native crystal structures were solved by molecular replacement with PHASER using the MAD structure as an initial model. There are two molecules in asymmetric unit. PyMOL was used to calculate the cavity and binding pocket and draw the structure. The human LSD1 SWIRM domain coordinates have been deposited in the Protein Data Bank (PDB Accession code: 5IT3).

### 2.14 Prediction of the interaction between LSD1 and NR2E1 LBD

ZDOCK V3.0.2 (http://zdock.umassmed.edu/) was used to predict the interaction between LSD1 (PDB code: 3ZMU) and NR2E1 LBD (PDB code: 4XAI) by rigid body docking.^26^ ZDOCK explicitly searches rotational space and uses a Fast Fourier Transformation (FFT) algorithm to speed up searching in translational spaces. Residues 256–333, 410–435, 575–626, 717–742 and 792–819 of LSD1, assumed to be not participating in the interaction between LSD1 and NR2E1 LBD were set as block residues. ZDOCK generated docking complexes were then clustered by the MMTSB clustering method (http://www.mmtsb.org) at 8 Å RMSD cutoff.^27^ LSD1 peptide accessible areas on NR2E1 were calculated as buried solvent‐accessible area with a 1.4 Å probe radius using the Naccess software.^28^

### 2.15 Luciferase Assay

Pten promoter containing LSD1 and NR2E1 binding site was cloned into pGL3‐basic plasmid to drive the expression of a luciferase gene. Renilla plasmid (5 ng/well) and pGL3‐basic plasmid (100 ng/well) or pGL3‐Pten promoter plasmid (100 ng/well) were co‐transfected into indicated cells using Lipofectamine 3000. The luciferase activity was examined by a Duo‐Lite™ Luciferase Assay System 48 h after transfection (Vazyme, DD1205) following the manufacturer's instructions. Each assay was repeated three times independently. The average values of pGL3‐Pten promoter plasmid transfected samples were normalized with the values of PGL3‐basic plasmid transfected samples and the Renilla activity.

### 2.16 Acute toxicity assessment of peptides on mouse

Following peptides were synthesized in the Genscript Biotech Corporation (China).

TAT‐GFP: AYGRKKRRQRRRMDYKDHDGDYKDHDIDYKDDDDKRSMVSKGEELFTGVVPIL;TAT‐LSD1‐197‐211:AYGRKKRRQRRRMDYKDHDGDYKDHDIDYKDDDDKRSMPDIISGPQQTQKVFL. 100 μg TAT‐LSD1‐197‐211 peptide or TAT‐GFP peptide in 0.2 ml saline solution was administrated to 6 to 8‐week‐old female nude mouse by introveinous injection. The mice were monitored for 14 days and then sacrificed by CO_2_ inhalation. The mouse weight was measured. Mouse blood was obtained by retro‐orbital blood collection and then centrifuged to collect serum. The level of ALT, AST, CR and CKMB in serum was measured by a biochemical analyser (Wuhan Servicebio Technology Co., Ltd. China). Organs of mice were harvested and embedded in paraffin blocks. The block was sectioned for Hematoxylin and Eosin (HE) staining. The photos were taken with Slide scanning image system machine (Shenzhen Shengqiang Technology Co., Ltd., China).

## 3 RESULTS

### 3.1 NR2E1 and LSD1 are essential for the proliferation of BTICs

Brain tumour initiating cells (BTIC‐1 and BTIC‐2), also named brain tumour stem cells were derived from Nestin‐TV‐a mice and characterized by Prof. Haikun Liu's lab.^3^ We received these cells as a gift from Prof. Liu and maintained them in monolayer or non‐adherent suspension culture as reported^3^ (Figure S1A). These BTICs formed tumour spheres when cultured in suspension. Immunostaining of the tumour sphere revealed strong expression of NESTIN and NR2E1, the neural stem cell markers (Figure S1B).^3^ Treatment of BTICs by retinoic acid (RA) led the cells to differentiate into GFAP positive astrocytes and TUJ1 positive neuronal cells (Figure S1C,D). All these results confirm the stem cell property of BTICs.

**FIGURE 1:**
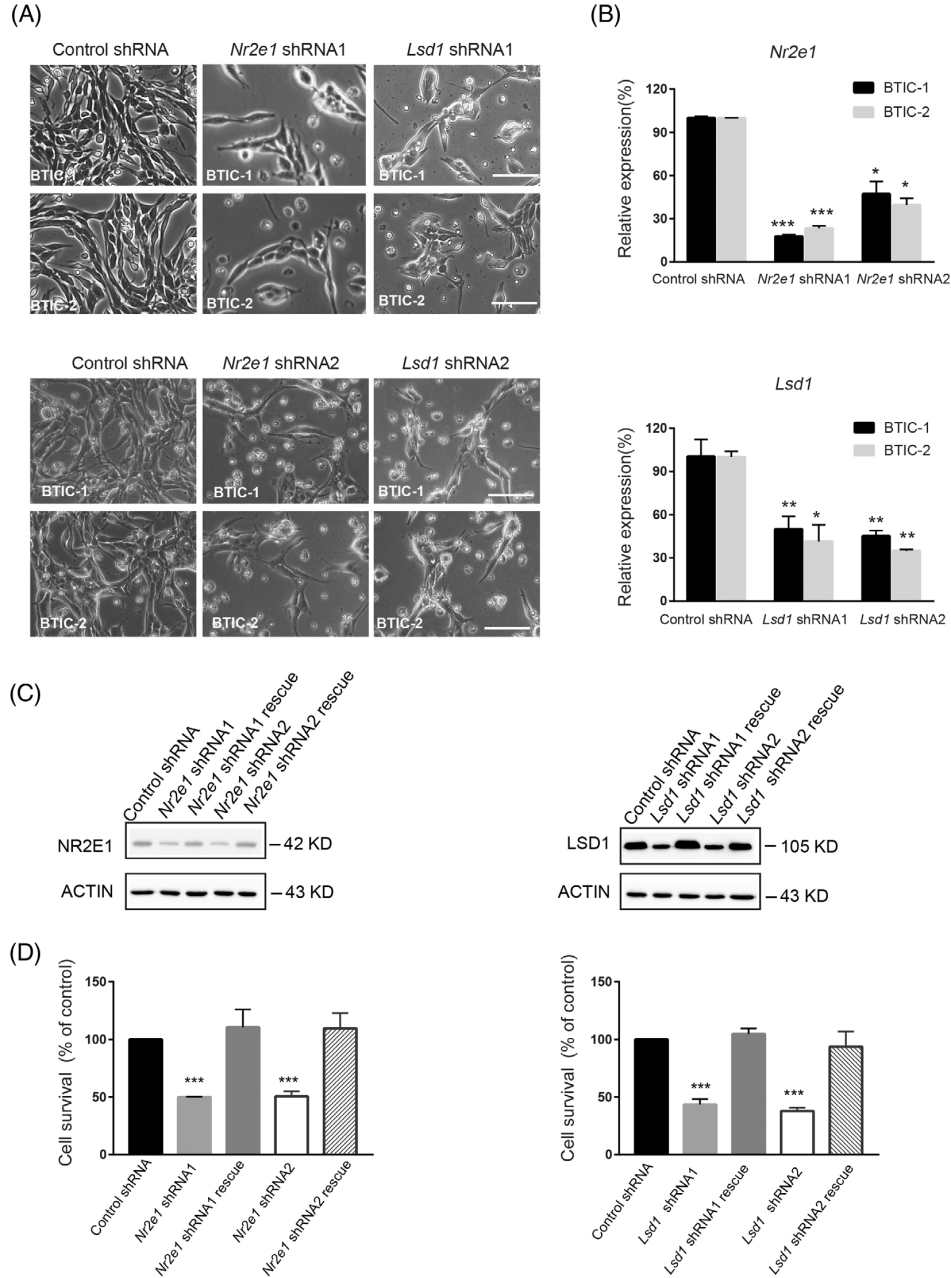
NR2E1 and LSD1 are required for the proliferation of BTICs. (A) Cell morphology of BTICs after knockdown of *Nr2e1* or *Lsd1* by RNAi. The scale bar is 100 μm. (B) Real‐time PCR analysis of mRNA level of *Nr2e1* and *Lsd1* in BTIC at day 3 after control shRNA, *Nr2e1* shRNA or *Lsd1* shRNA knockdown. Data are represented as mean ± SD (*n* = 3). Statistically significant differences, calculated through Student's *t*‐test, are indicated (*p < 0.05; **p < 0.01; ***p < 0.001). (C) Representative western blot result of the protein level of NR2E1 and LSD1 in BTICs at day 3 after transfected with control shRNA, *Nr2e1* shRNA, *Lsd1* shRNA or shRNA together with its respective RNAi‐immune cDNA as indicated. ACTIN was used as an internal control. (D) MTT assay of the cell viability of BTICs at day 3 after transfected with control shRNA, *Nr2e1* shRNA, *Lsd1* shRNA or shRNA together with its respective RNAi‐immune cDNA as indicated. Data are represented as mean ± SD (*n* = 3). Statistically significant differences, calculated through Student's *t*‐test, are indicated (*p < 0.05; **p < 0.01; ***p < 0.001)

To study the role of NR2E1 and LSD1 in BTICs, we designed two different shRNAs for each gene and cloned them into pSuper‐puro vector. These shRNAs were transfected into BTICs respectively by electroporation and the transfected cells were selected with puromycin. Three days after puromycin selection, *Nr2e1* and *Lsd1* shRNA knockdown led to a significantly lower amount of BTICs in culture (Figure 1A). Real‐time PCR revealed that the expression of *Nr2e1* and *Lsd1* was downregulated more than 50% of their original levels by their respective shRNAs (Figure 1B). To ensure the specificity of the shRNA, we generated rescue cDNA by making silence mutations at the shRNA targeted cDNA region and co‐transfected the mutated cDNA and its respective shRNA into BTICs. Western blot revealed that the expression of NR2E1 and LSD1 could be efficiently knocked down by their respective shRNA and then rescued by shRNA specific rescue cDNA (Figure 1C), confirming the specificity of shRNA. Cell proliferation assay also revealed that the reduced cell viability caused by *Nr2e1* and *Lsd1* shRNA could be rescued by expressing the shRNA rescue cDNA (Figure 1D).

To understand whether *Nr2e1* or *Lsd1* knockdown caused cell viability reduction is due to reduced cell proliferation or increased apoptosis, we first performed BrdU incorporation assay. We found that BrdU positive cells were significantly reduced after *Nr2e1* or *Lsd1* knockdown, indicating that *Nr2e1* and *Lsd1* are required for the proliferation of BTICs (Figure S1E). Next, we examined the expression of apoptosis marker—cleaved CASPASE 3 by western blot. *Nr2e1* or *Lsd1* knockdown led to the increment of cleaved CASPASE 3, suggesting an increase of apoptosis after *Nr2e1* and *Lsd1* knockdown (Figure S1F). Hence, the reduced cell viability caused by *Nr2e1* or *Lsd1* knockdown is a combined effect of reduced cell proliferation and enhanced apoptosis.

### 3.2 NR2E1 and LSD1 synergistically repress PTEN to promote BTIC proliferation

**FIGURE 2:**
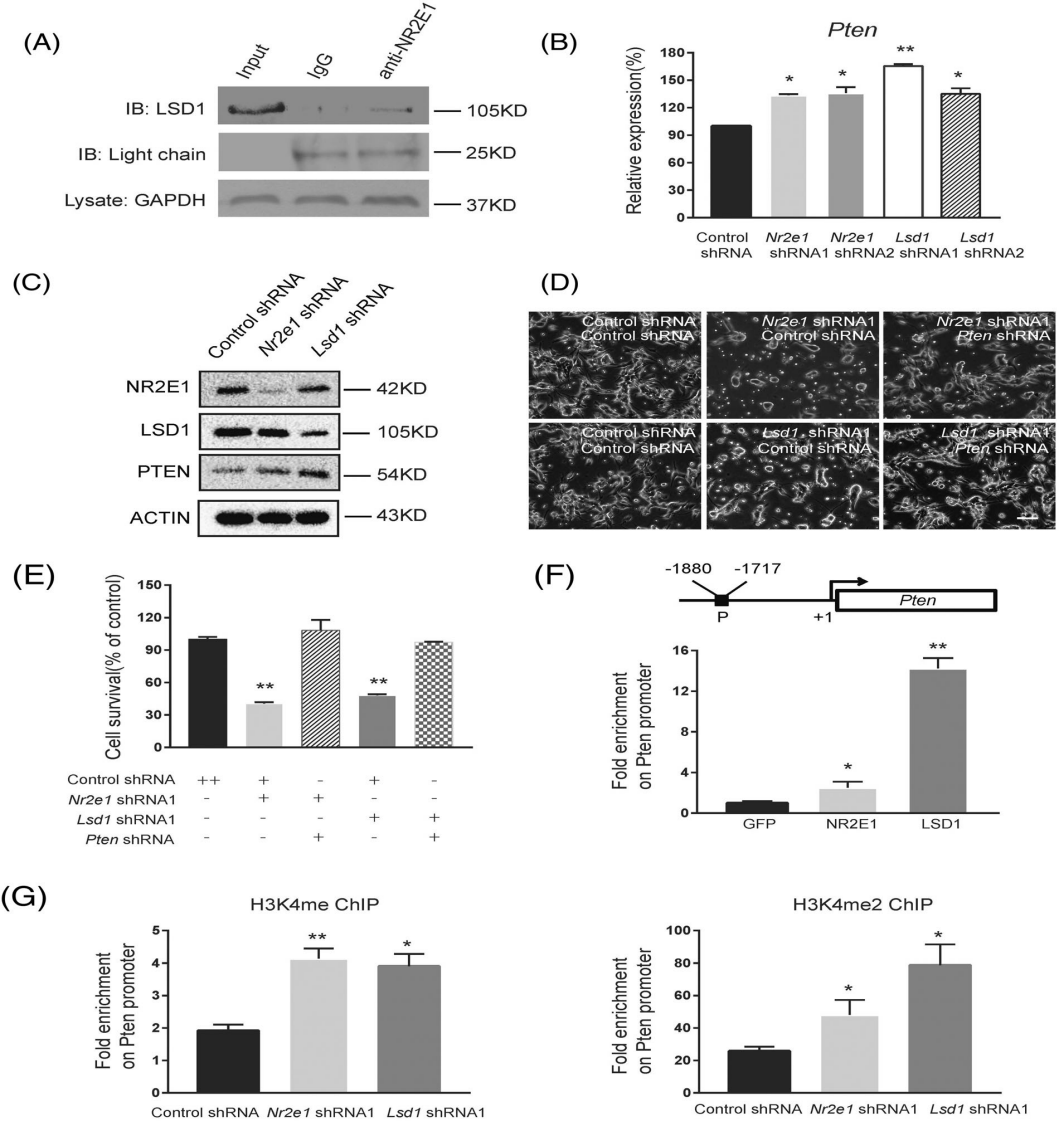
NR2E1 and LSD1 form a complex in BTICs and regulate Pten expression. (A) Co‐immunoprecipitation (Co‐IP) assay with antibodies against NR2E1 and LSD1 to test their interaction in BTICs. Equal amount of total cell lysate was immunoprecipitated with control IgG and an antibody against NR2E1. The pulled down proteins were then blotted with an anti‐LSD1 antibody. GAPDH in the lysate was shown as a control for the input material for each Co‐IP assay. IgG light chain was shown as a loading control of Co‐IP pull‐down material. (B) Real‐time PCR analysis of mRNA level of *Pten* in BTICs after control shRNA, *Nr2e1* shRNA or *Lsd1* shRNA knockdown. Data are represented as mean ± SD (*n* = 3). Statistically significant differences, calculated through Student's *t*‐test, are indicated (*p < 0.05; **p < 0.01). (C) Western blot analysis of protein level of NR2E1, LSD1, PTEN at 40‐h time point after control shRNA, *Nr2e1* shRNA1 or *Lsd1* shRNA1 knockdown. ACTIN was used as an internal control. (D) Cell morphology of BTICs after control shRNA, *Nr2e1* shRNA1, *Lsd1* shRNA1 and *Pt*en shRNA knockdown as indicated combination. Scale bar represents 100 μm. (E) Cell survival analysis of BTICs after control shRNA, *Nr2e1* shRNA1, *Lsd1* shRNA1 and *Pten* shRNA knockdown as indicated combination using MTT assay. Data are represented as mean ± SD (*n* = 3). Statistically significant differences, calculated through Student's *t*‐test, are indicated (*p < 0.05; **p < 0.01). (F) Scheme depicting the region where primers were designed for amplification of Chromatin immunoprecipitation (ChIP) DNA and relative enrichment of control GFP, LSD1 and NR2E1 at *Pten* promoter revealed by ChIP and real‐time PCR assay. Data are represented as mean ± SD (*n* = 3). Statistically significant differences, calculated through Student's *t*‐test, are indicated (*P < 0.05; **P < 0.01). (G) ChIP and real‐time PCR analysis of H3K4me and H3K4me2 enrichment at *Pten* promoter in BTICs after knockdown by control shRNA, *Nr2e1* shRNA or *Lsd1* shRNA at 40‐h time point. Data are represented as mean ± SD (*n* = 3). Statistically significant differences, calculated through Student's *t*‐test, are indicated (*p < 0.05; **p < 0.01)

It is reported that NR2E1 interacts with LSD1 and promotes neural stem cell proliferation via repressing *Pten* expression by demethylating H3K4me and H3K4me2 at *Pten* promoter.^18^ To examine whether the same mechanism was adopted in BTICs, we first performed co‐immunoprecipitation assay using the whole cell lysate. An anti‐NR2E1 antibody could pull down endogenous LSD1, but control IgG could not, suggesting that NR2E1 and LSD1 form a complex in BTICs (Figure 2A). Next, we examined the expression of PTEN in *Nr2e1* and *Lsd1* knockdown BTICs. The downregulation of NR2E1 and LSD1 led to the upregulation of PTEN at both mRNA and protein levels (Figure 2B,C). This is in line with the luciferase activity of Pten promoter being upregulated in Nr2e1 knockdown, Lsd1 knockdown as well as Nr2e1 and Lsd1 double knockdown BTICs (Figure S2A).

To examine whether PTEN is one of the major downstream effectors of NR2E1 and LSD1, we adopted Sigma approved shRNA of *Pten* for the rescue assay. This shRNA alone could efficiently downregulate *Pten* expression (Figure S2B,C). Downregulation of *Pten* by shRNA led to increased cell viability (Figure S2D). We then co‐transfected *Pten* shRNA with *Nr2e1* shRNA or *Lsd1* shRNA respectively into BTICs. We found that the reduced cell viability caused by *Nr2e1* or *Lsd1* knockdown could be rescued by knockdown of *Pten* (Figure 2D,E, Figure S2E,F), suggesting that PTEN is a major downstream effector of NR2E1 and LSD1 in regulating BTIC viability. Further chromatin immunoprecipitation (ChIP) assay revealed that NR2E1 and LSD1 both bind at the promoter of *Pten* (Figure 2F). Using antibodies against H3K4me and H3K4me2 to pull down chromatin extracted from *Nr2e1* or *Lsd1* knockdown BTICs, we found that both downregulation of NR2E1 and LSD1 led to increased enrichment of H3K4me and H3K4me2 at the *Pten* promoter, suggesting that NR2E1 and LSD1 indeed directly repress *Pten* in BTICs by demethylating H3K4me and H3K4me2 at its promoter (Figure 2G).

Therefore, our data proved that BTICs adopted the same NR2E1/LSD1‐Pten regulatory axis as neural stem cells.

### 3.3 Prediction of LSD1 peptides involved in the NR2E1‐LSD1 interaction

**FIGURE 3:**
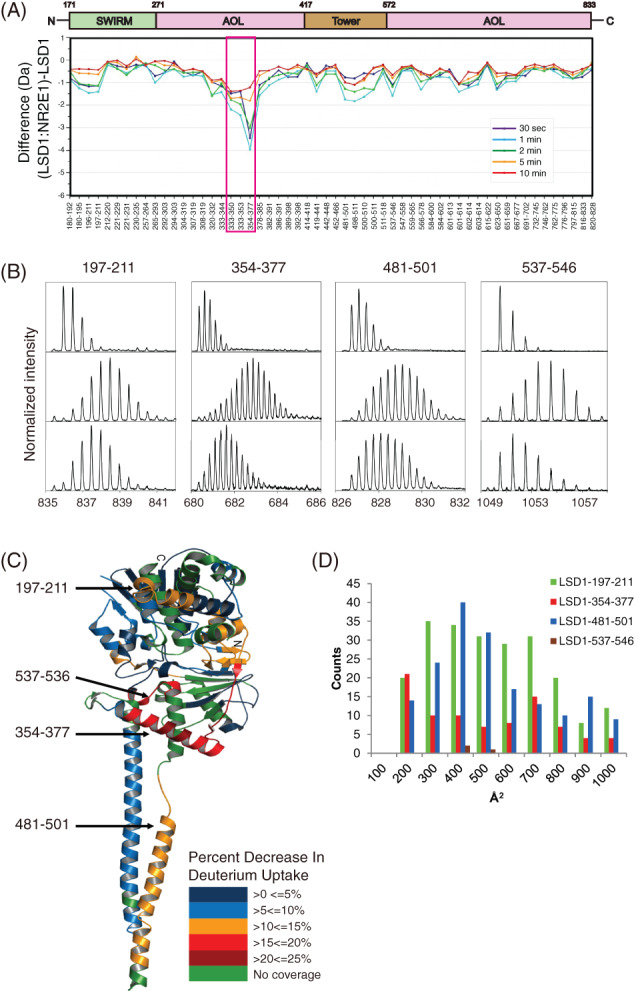
Effect of NR2E1 on LSD1 as shown by HDX‐MS. (A) The difference in absolute deuterium uptake between LSD1 and NR2E1:LSD1. (B) Enhanced mass spectra for four pepsin digest fragments of LSD1, 197–211, 354–377, 481–501 and 537–546 which show significant differences in exchange upon NR2E1 binding: undeuterated peptide (Top), isotopic envelope for the same peptide from LSD1 alone following 1 min deuteration (Middle) and isotopic envelope for the same peptide from LSD1 in complex with NR2E1 following 1 min deuteration (Bottom). (C) Heat map showing the percent decrease in deuterium uptake for NR2E1:LSD1 relative to LSD1 following 1 min of HDX, mapped on the crystal of LSD1 (PDB ID: 2Z3Y). (D) The histogram plot of the distribution of the surface area of NR2E1 buried by LSD1 peptides 197–211, 354–377, 481–501 and 537–546. (Buried surface area less than 100Å^2^ is neglected)

To understand how NR2E1 and LSD1 synergistically function, we employed Amide Hydrogen/Deuterium Exchange and Mass Spectrometry (HDX‐MS) to investigate the interaction between NR2E1 and LSD1. A total of 56 pepsin digested fragments covering about 80% of the LSD1 primary sequence were identified and analysed. The difference in deuterium uptake for all the fragments between LSD1 alone and LSD1:NR2E1 complex was measured at 30 s, 1, 2, 5 and 10 min. As the difference in deuterium exchange was maximum at 1 min, the deuterium uptake for each peptide in the 1 min samples was used to monitor the effects of NR2E1 binding with LSD1. A number of regions in LSD1 showed decreased exchange upon interactions with NR2E1, but the maximum difference occurred at the regions within the AO domain. Peptides 333–350, 333–353 and 354–377 from the AO domain showed a significant decrease of 2.2, 2.5 and 4.0 deuterons, respectively (Figure 3A). Also, in the LSD1:NR2E1 complex, peptides 196–211, 197–211, 320–332, 333–344, 378–385, 419–441, 481–501, 498–511, 500–510, 537–546, 601–614 and 623–650 from the SWIRM, Tower and AO domains showed a decrease of about 1.6 deuterons. Mass spectral isotope envelopes for four peptides, 197–211, 354–377, 481–501 and 537–546, from 1 min HDX samples showed the most significant difference between LSD1 alone and NR2E1‐LSD1 complex after deuterium uptake, suggesting these LSD1 peptides may be involved in forming a complex with NR2E1 (Figure 3B). The difference in deuterium uptake for each peptide was calculated and the results from 1 min samples were mapped onto the crystal structure of LSD1 (PDB ID: 2Z3Y) (Figure 3C).

The crystal structure of the NR2E1 ligand binding domain (LBD) has been solved.^29^ NR2E1 LBD interacts with both the SWIRM and AO domains of LSD1.^10^ We have predicted the interaction between NR2E1 LBD (PDB code: 4XAI) and LSD1 (PDB code: 3ZMU) using the ZDOCK program.^26^ LSD1 residues 256–333, 410–435, 575–626, 712–744 and 792–819 that are not supposed to interact with NR2E1 were set as block residues. As a result, 3600 docking complexes were generated and clustered into 650 groups using the MMSTB clustering method^27^ with a root‐mean‐square deviation (RMSD) cutoff at 8 Å. The buried solvent‐accessible area, which is deemed as the possible binding surface of NR2E1 LBD by LSD1 peptides 197–211, 354–377, 481–501 and 537–546, was calculated by Naccess^28^ with a probe radius of 1.4 Å (Table S2). LSD1 peptides 197–211, 354–377 and 481–501 individually showed varying predicted binding surface on NR2E1. However, LSD1‐537‐546 showed almost no predicted binding surface, suggesting that LSD1‐537‐546 is not likely involved in the interaction between LSD1 and NR2E1 LBD (Figure 3D).

### 3.4 Role of LSD1 peptides in NR2E1‐LSD1 interaction

**FIGURE 4:**
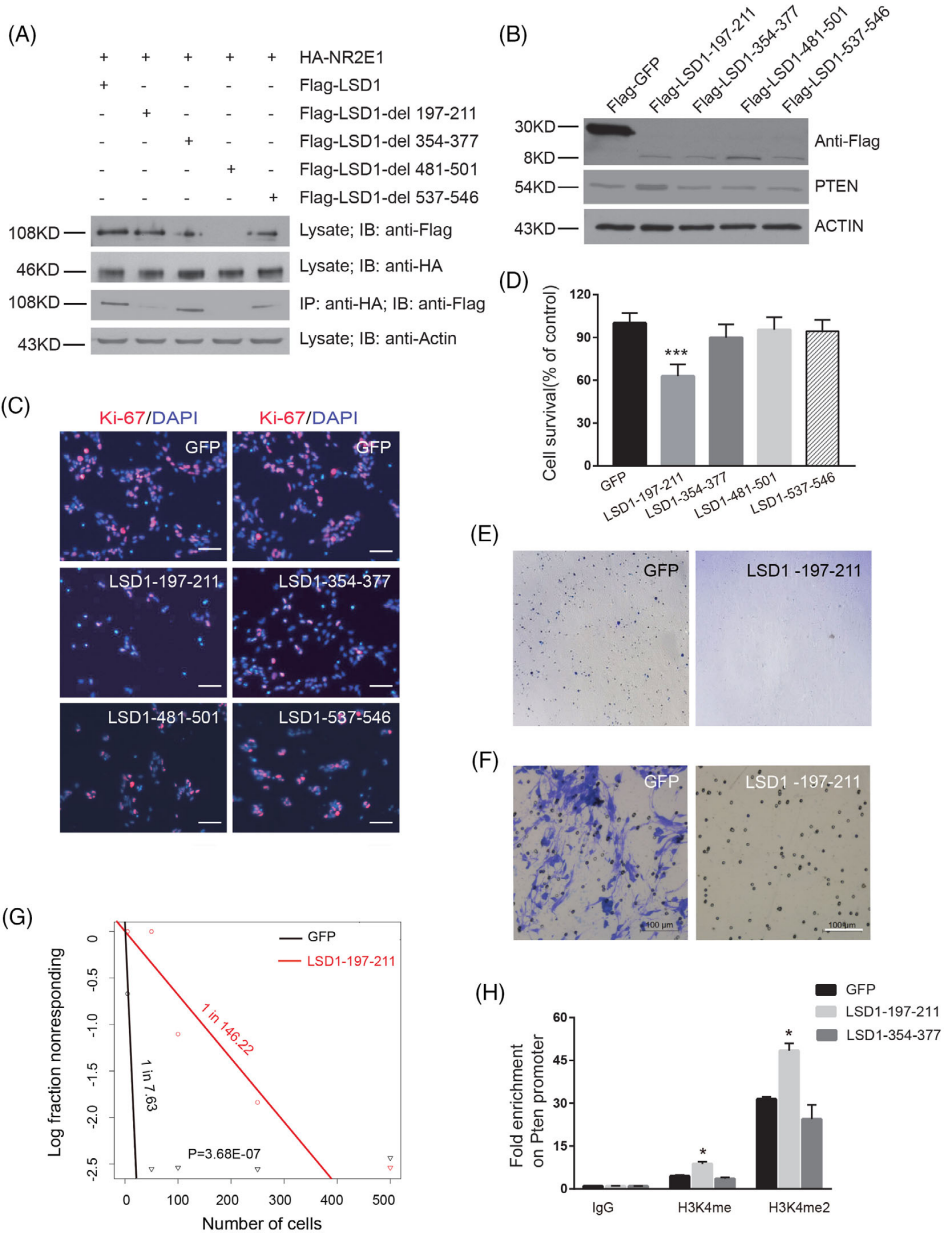
The role of LSD1 peptides on NR2E1 and LSD1 interaction. (A) The interaction between NR2E1 and LSD1 was disrupted by the deletion of residues 197–211 in LSD1. Total protein lysate of HA‐NR2E1 and Flag‐LSD1 or Flag‐LSD1 mutant transfected 293T cells were immunoprecipitated with anti‐HA antibody, followed by immunoblotting with anti‐Flag antibody. Protein expression in the cell was shown by immunoblotting with anti‐Flag or anti‐HA antibody. (B) Overexpression of Flag‐LSD1 peptide 197–211 led to the upregulation of PTEN in BTICs. Lysates of Flag‐GFP and Flag‐LSD1 peptide overexpressed BTICs were immunoblotted by anti‐Flag and anti‐PTEN antibodies. ACTIN was loaded as an internal loading control. (C) Overexpression of LSD1 peptide 197–211 led to a reduction of Ki‐67 positive cells. Anti‐Ki‐67 antibody staining was shown in red and DAPI staining was shown in blue. The scale bar is 25 μm. (D) MTT assay of the viable BTICs after transient overexpression of control or LSD1 peptides. Data are represented as mean ± SD (*n* = 6). Statistically significant differences, calculated through Student's *t*‐test, are indicated (***p < 0.001). (E) Representative picture of soft agar assay of BTICs after overexpression of control peptide or LSD1‐197‐211 peptide. (F) Representative picture of transwell assay to show the migration capacity of GFP overexpressed BTICs and LSD1‐197‐211 overexpressed BTICs. Scale bar represents 100 μm. (G) Limiting dilution assay of GFP control or LSD1‐197‐211 overexpressed BTICs. (H) Relative enrichment of H3K4me and H3K4me2 on *Pten* promoter after overexpression of GFP, LSD1‐197‐211 peptide or LSD1‐354‐377 peptide in BTICs. Data are represented as mean ± SD (*n* = 3). Statistically significant differences, calculated through Student's *t*‐test, are indicated (*p < 0.05)

To further characterize the role of the LSD1 peptides 197–211, 354–377, 481–501 and 537–546 identified by HDX‐MS, we generated Flag‐tagged *Lsd1* mutant clones by deleting the peptide encoding regions. These clone plasmids were then co‐transfected with a plasmid expressing HA‐NR2E1 into 293T cells for co‐immunoprecipitation assay. Western blot revealed that while Flag‐LSD1‐∆197–211, Flag‐LSD1‐∆354–377 and Flag‐LSD1‐∆537–546 could be stably expressed, deletion of residues 481–501 of LSD1 however led to no detectable protein, suggesting this region is critical for the stable expression of LSD1 (Figure 4A). Then we performed immunoprecipitation with an anti‐HA antibody using whole cell lysate and followed by immunoblotting with an anti‐Flag antibody. It turned out that HA‐NR2E1 could pull down Flag‐LSD1, Flag‐LSD1‐∆354–377 and Flag‐LSD1‐∆537–546, but not Flag‐LSD1‐∆197–211. This suggests that deletion of peptide LSD1‐354‐377 or LSD1 537–546 did not disturb the interaction between LSD1 and NR2E1, only deletion of LSD1‐197‐211 destroyed the NR2E1‐LSD1 complex (Figure 4A). Therefore, LSD1‐197‐211 is essential for NR2E1‐LSD1 complex formation.

We next investigated the function of overexpression of these peptides in BTICs. We cloned the four peptides into the pCAG‐puro plasmids and expressed them as Flag‐tag peptides. Twelve hours after transfection, puromycin was added to select the transfected cells. On day 3 after puromycin selection, cells were harvested to check protein expression. Western blot with an anti‐Flag antibody confirmed that all four Flag‐tagged LSD1 peptides were expressed. Interestingly, the level of PTEN was increased in Flag‐LSD1‐197‐211 overexpressed BTICs compared to GFP overexpressed BTICs (Figure 4B). Immunostaining BTICs at this stage with an anti‐Ki67 antibody revealed that overexpression of LSD1‐197‐211 led to fewer Ki67 positive cells (Figure 4C). Further MTT assay confirmed that LSD1‐197‐211 transfected BTICs showed the most drastic reduction of viable cells (Figure 4D).

As transient expression of LSD1‐197‐211 could inhibit the self‐renewal of BTICs, we next generated doxycycline inducible lentivirus to stably express LSD1‐197‐211‐P2A‐EGFP in BTICs. LSD1‐197‐211 peptide and EGFP are connected with P2A, a self‐cleavage peptide (Figure S3A). After translation, LSD1‐197‐211 and EGFP are cut apart at P2A site. Hence, GFP protein level can reflect LSD1‐197‐211 level in transduced cells. We first infected 293T cells with these lentiviruses. After doxycycline induction for 3 days, it is clearly that both GFP lentivirus and LSD1‐197‐211‐GFP lentivirus express similar level of GFP. Therefore, LSD1‐197‐211 did not inhibit the growth of 293T cells (Figure S3B). Next, we infected BTICs with the same batch of lentivirus and purified the transduced cells by puromycin selection. We then induced the peptide expression with doxycycline. Sixty hours after induction, we performed immunostaining with antibody against cleaved CASPASE‐3, the marker of apoptosis. It turned out that BTICs that expressed LSD1‐197‐211 and GFP were often cleaved CASPASE‐3 positive, while it was not the case for the control GFP overexpressed BTICs (Figure S4A). This result suggests that the expression of LSD1‐197‐211 in BTICs resulted in apoptosis of the cells. Further extending the cell culture time, we observed that GFP positive LSD1‐197‐211 BTICs gradually lost their shape and detached from the plate. Eventually, very rare GFP positive cells in LSD1‐197‐211 lentivirus transduced BTIC culture were observed (Figure S4B). Analysis of GFP positive cell ratio with flow cytometry using doxycycline induced lentivirus freshly transduced BTICs revealed that in contrast to the 78.6% GFP positive cells in the GFP lentivirus transduced BTICs, only 0.71% of the LSD1‐197‐211 lentivirus transduced BTICs showed weak GFP expression, suggesting that LSD1‐197‐211 peptide expression eliminated BTICs (Figure S4C). Soft agar colony formation assay also revealed that LSD1‐197‐211 overexpression drastically reduced the colony formation capacity of BTICs (Figure 4E). Furthermore, transwell assay revealed that overexpression of LSD1‐197‐211 drastically reduced the migration capacity of BTICs (Figure 4F). As expected, the size of tumour spheres formed by LSD1‐197‐211 overexpressed BTICs was smaller than GFP overexpressed BTICs (Figure S4D). Besides, the overall sphere number was also much less in LSD1‐197‐211 overexpressed BTICs than GFP overexpressed BTICs (Figure S4E). Limiting dilution assay revealed drastic decrease in tumour sphere formation rate in LSD1‐197‐211 overexpressed BTICs (Figure 4G). Hence, LSD1‐197‐211 efficiently inhibits the growth of BTICs in vitro.

To examine whether LSD1‐197‐211 can really interfere NR2E1 and LSD1 interaction, we co‐expressed HA‐NR2E1, Flag‐LSD1 and LSD1‐197‐211 peptide in 293T cells and then performed pull‐down assay. We found that the pulldown efficiency of HA‐NR2E1 by Flag‐LSD1 was dramatically reduced by overexpression of LSD1‐197‐211 peptide. Reciprocal pulldown of Flag‐LSD1 by HA‐NR2E1 was also interfered by LSD1‐197‐211 peptide (Figure S4F). We further performed ChIP assay with antibodies against H3K4me and H3K4me2 with chromatin extracted from GFP, LSD1‐197‐211 and LSD1‐354‐377 overexpressed BTICs respectively. H3K4me and H3K4me2 modification at *Pten* promoter was significantly increased in LSD1‐197‐211 overexpressed BTICs, compared to GFP overexpressed BTICs and LSD1‐354‐377 overexpressed BTICs (Figure 4H). These data suggest that LSD1‐197‐211 can inhibit BTIC proliferation by interfering the demethylation function of NR2E1‐LSD1 complex on H3K4me and H3K4me2 at *Pten* promoter.

### 3.5 Specificity of peptide LSD1‐197‐211

**FIGURE 5:**
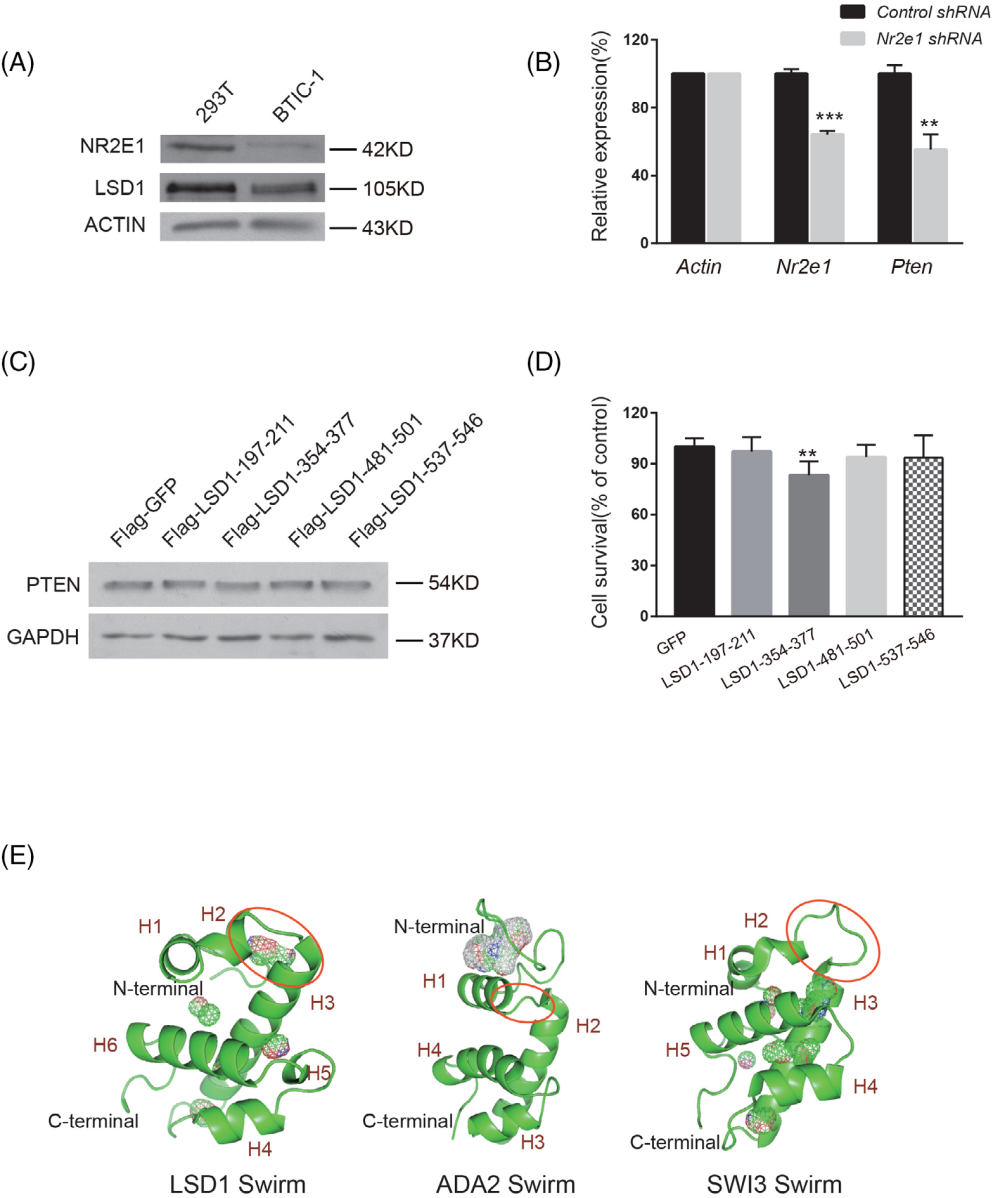
Specificity of LSD1‐197‐211. (A) Western blot to check the protein level of NR2E1 and LSD1 in 293T and BTIC‐1. ACTIN was loaded as an internal control. (B) Real‐time PCR quantification of the mRNA levels of *Pten* and *Nr2e1* in control shRNA and *Nr2e1* shRNA knockdown 293T cells. The data were normalized with *Actin*. Data are represented as mean ± SD (*n* = 3). Statistically significant differences, calculated through Student's *t*‐test, are indicated (**p < 0.01; ***p < 0.001). (C) Western blot to compare the expression level of PTEN in different LSD1 peptide overexpression 293T cells and control GFP overexpression 293Ts cells. GAPDH was loaded as an internal control. (D) MTT assay of the cell viability of 293T cells after overexpression with control or LSD1 peptides. Data are represented as mean ± SD (*n* = 6). Statistically significant differences, calculated through Student's *t*‐test, are indicated (**p < 0.01). (E) Overall structures of LSD1 SWIRM, ADA2 SWIRM (PDB Id: 2AQE) and SWI3 SWIRM (PDB ID: 2FQ3). All SWIRM domains contain a central long helix, flanked by small helices. LSD1‐197‐211 and the corresponding region in the ADA2 and SWI3 SWIRM domains are circled. Cavity/binding pocket is represented as mesh map. The figures are drawn with PyMOL

Although LSD1‐197‐211 could interfere with the synergistic function of NR2E1 and LSD1 and inhibit the proliferation of BTICs, the specificity of this peptide is unclear. Both NR2E1 and LSD1 are highly expressed in 293T cells (Figure 5A). Knockdown of *Nr2e1* by shRNA did not, however, lead to the upregulation of *Pten* at the mRNA level, suggesting that the NR2E1‐LSD1 mechanism is not involved in the proliferation of 293T cells (Figure 5B). To test whether LSD1‐197‐211 had any effect on the cells that do not rely on the NR2E1‐LSD1 based cell proliferation, we overexpressed GFP and LSD1 peptides in 293T cells separately with puromycin selection for 3 days. Western blot showed that the PTEN protein level was similar in the GFP and LSD1 peptide overexpressed 293T cells (Figure 5C). MTT assay was further performed to check the cell viability. Apart from LSD1‐354‐377, which exhibited slight inhibition of 293T cell growth, other peptides showed no obvious inhibitory effect (Figure 5D). Besides 293T cells, we also examined whether LSD1‐197‐211 peptide had any effect in glioma cell lines LN299, T98G and U251. CCK8 assays revealed that LSD1‐197‐211 peptide could not inhibit these cells (Figure S5A). These results suggest that the LSD1‐197‐211 peptide selectively inhibits BTICs.

To further characterize the specificity of LSD1‐197‐211, we determined the crystal structure of the human LSD1 SWIRM domain, residues 183–267 (Table S1). The SWIRM structure mainly contains a long central helix separating two smaller helix–loop–helix motifs at both sides (Figure 5E). The SWIRM domain highly resembles the SWIRM domain of previously determined human LSD1 crystal structure (PDB ID: 2Z3Y) (Figure S5B). Out of 687 aligned atoms, 528 atoms of the two structures can be well aligned with a root mean square of 0.418. Unlike the SWIRM domain of SWI3 and ADA2, which binds to DNA, the SWIRM domain of LSD1 can neither bind to DNA nor does it contain any typical DNA‐binding patch.^30^ One major difference between LSD1 SWIRM domain and SWI3 and ADA2 SWIRM domains is mainly confined to the N‐terminus. LSD1‐197‐211 is located at this region of the SWIRM domain, comprising a part of helix H2, a loop and a part of the long central helix H3 (Figure 5E). This region protrudes away from the hydrophobic core formed by H6 and the AO domain, which is involved in substrate demethylation.^31^ It forms a stable binding pocket with the N terminal loop and provides enough space to interact with other proteins (Figure 5E). Unlike LSD1 SWIRM domain, the corresponding region of ADA2 SWIRM is blocked by the N‐terminal loop, making it impossible to interact with other proteins. For the SWI3 SWIRM domain, a big loop connects the H2 and H3 helices and no binding pocket is formed in this region (Figure 5E). The structural difference among the SWIRM domains of LSD1, ADA2 and SWI3 substantiates the unique ligand binding property of LSD1 SWIRM domain.

### 3.6 LSD1 197–211 inhibits the brain tumour formation of human BTIC

Human and mouse NR2E1 protein sequences share more than 97% similarity, so does LSD1. In addition, human and mouse LSD1‐197‐211 peptide sequences are exactly the same. As compared to other histone H3K4 demethylase, LSD1 is highly expressed in BTICs (Figure S6A), manifesting its importance in BTICs. Hence, we deduced that LSD1‐197‐211 peptide should be able to repress human BTICs (hBTICs) as well. To test this hypothesis, we derived two hBTICs from surgical tissue of glioblastoma patients who were diagnosed of grade IV glioblastoma in Sun Yat‐sen University Cancer Center (Figure S6B). The cells expressed NESTIN and NR2E1, the neural stem cell markers (Figure S6C). They differentiated into GFAP positive astrocytes and TUJ1 positive neuronal cells by retinoid acid treatment (Figure S6D,E). Western blot further confirms that neural stem cell marker NESTIN and NR2E1 were highly enriched in mouse and human BTICs as compared to the glioma cell lines U251, T98G and LN229 (Figure S6F). Cancer stem cell marker CD133 was also expressed at a significantly higher level in BTICs than glioma cell lines (Figure S6G).

We then knocked down *Nr2e1* and *Lsd1* by shRNA in hBTICs. Western blot revealed that either NR2E1 or LSD1 downregulation led to the increment of PTEN protein (Figure S6H,I), indicating that hBTICs adopt the same regulatory mechanism as mouse BTICs.

**FIGURE 6:**
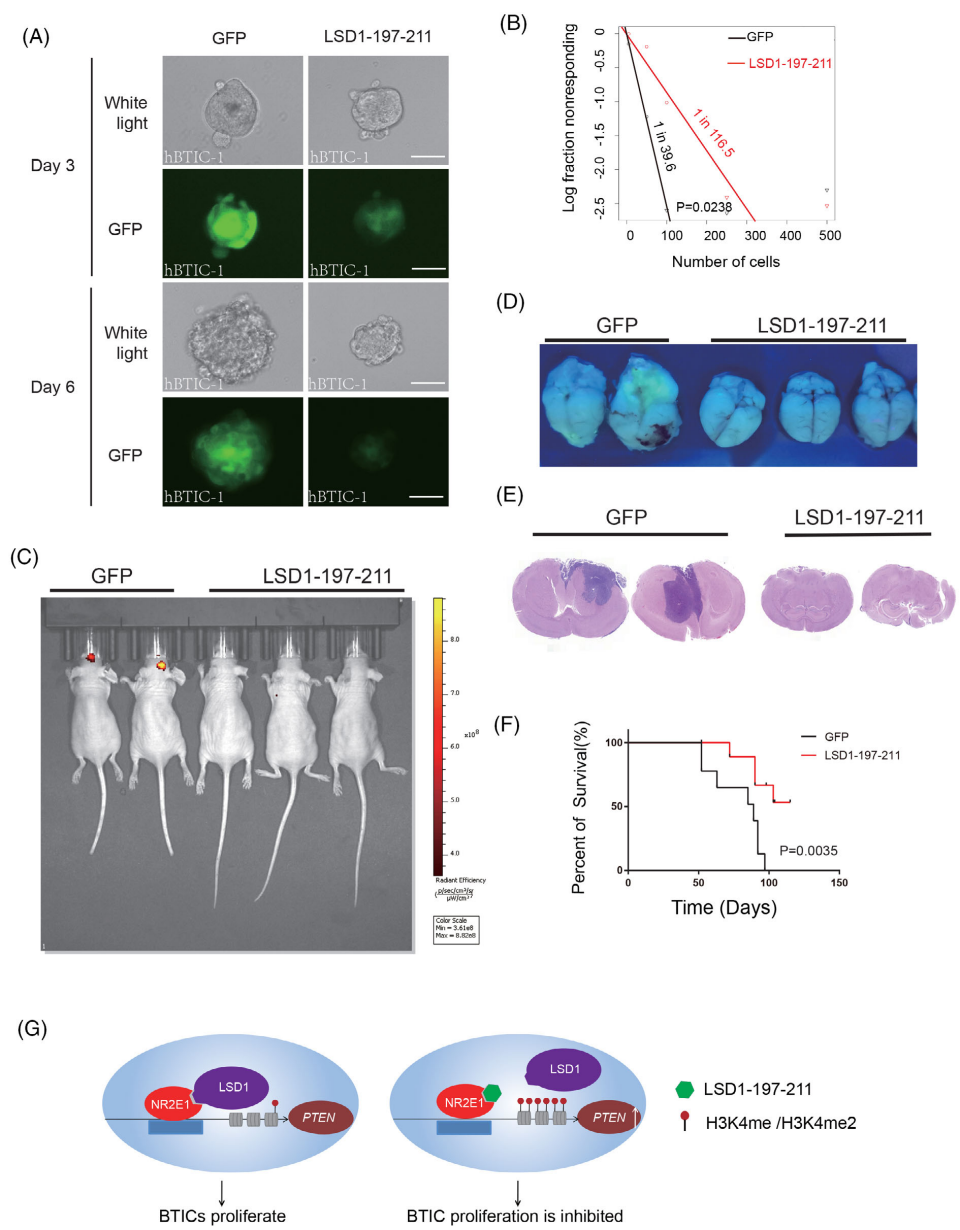
Stable expression of LSD1‐197‐211 inhibits the proliferation of human BTICs. (A) Representative pictures of suspension culture of human BTIC line 1 (hBTIC‐1) transduced by lentivirus that express GFP or LSD1‐197‐211‐GFP after induction by doxycycline for 3 days and 6 days. Light microscopy (Top), fluorescence microscopy to detect GFP (Bottom). Scale bar represents 50 μm. (B) Limiting dilution assay of GFP control or LSD1‐197‐211 overexpressed hBTICs. (C) Representative IVIS images of brain tumours in nude mice transplanted with GFP or LSD1‐197‐211‐GFP lentivirus transduced human BTICs after 5 weeks of doxycycline induction. (D) Brains of nude mice transplanted with GFP or LSD1‐197‐211‐GFP lentivirus transduced human BTICs under UV light. (E) Representative hematoxylin–eosin staining pictures of brains of nude mice transplanted with GFP or LSD1‐197‐211‐GFP lentivirus transduced human BTICs after 7 weeks of doxycycline induction. (F) Survival curve of nude mice transplanted with GFP or LSD1‐197‐211‐GFP lentivirus transduced hBTICs. *X*‐axis is the day after hBTIC transplantation. (G) A model to depict the functional mechanism of NR2E1, LSD1 and LSD1‐197‐211 in BTICs

We next generated doxycycline inducible LSD1‐197‐211‐GFP and control GFP transduced hBTICs by above mentioned lentivirus system. Western blot revealed that doxycycline induced LSD1‐197‐211 expression led to increased expression of PTEN in hBTICs (Figure S6J). The colony formation capacity of hBTICs was also significantly inhibited by doxycycline induced LSD1‐197‐211 expression (Figure S6K). In addition, 3 days after doxycycline induction, tumour spheres formed by LSD1‐197‐211‐GFP transduced hBTICs were obviously smaller than tumour spheres formed by GFP transduced hBTICs. GFP signal in LSD1‐197‐211‐GFP transduced hBTICs was also weaker than GFP transduced hBTICs. Six days later, the difference was more drastic (Figure 6A and Figure S7A). Not only the number of tumour spheres formed by LSD1‐197‐211‐GFP transduced hBTICs was less than GFP transduced hBTICs, but also the average size of tumour spheres formed by LSD1‐197‐211‐GFP transduced hBTICs was smaller than tumour spheres formed by GFP transduced hBTICs (Figure S7B,C). Limiting dilution assays also revealed decreased tumour sphere formation rate in LSD1‐197‐211 overexpressed BTICs (Figure 6B). These results confirmed that LSD1‐197‐211 inhibits hBTICs.

To further investigate the function of LSD1‐197‐211 in vivo, we transplanted the doxycycline inducible GFP transduced hBTICs or LSD1‐197‐211‐GFP transduced hBTICs into the brains of nude mice respectively. The next day after the transplantation, we started to feed the mice with water or water containing doxycycline to induce transduced gene expression. For the group of water without doxycycline, mice transplanted with either GFP transduced hBTICs or LSD1‐197‐211‐GFP transduced hBTICs became skinny. Hematoxylin–eosin (HE) staining of mouse brain confirmed tumour formation (Figure S7D,E). When the mice were fed with water containing doxycycline, GFP positive brain tumour could be detected by Xenogen IVIS in mice transplanted with GFP transduced hBTICs 5 weeks later, while little or no GFP positive signals could be detected in mice transplanted with LSD1‐197‐211‐GFP transduced hBTICs (Figure 6C, Figure S7F). When the mouse brains were harvested, only mouse brain with GFP transduced hBTIC transplantation showed visible GFP fluorescence (Figure 6D). Further hematoxylin–eosin staining revealed obvious tumour infiltration in the brains of GFP transduced hBTIC transplanted mice, while most brains of mice transplanted with LSD1‐197‐211‐GFP transduced hBTICs showed either no or tiny tumour infiltration (Figure 6E). The mean tumour size of LSD1‐197‐211‐GFP transduced hBTIC formed brain tumour was significantly smaller than GFP transduced hBTIC formed brain tumours (Figure S7G). The type of transplanted hBTICs was double confirmed by PCR using mouse brain tissue (Figure S7H). LSD1‐197‐211 expression showed obviously beneficial effect to hBTIC transplanted mice. The body weight of control BTIC transplanted mice was greatly reduced compared to LSD1‐197‐211‐GFP hBTIC transplanted mice at week seven after doxycycline induction (Figure S7I,J). Mice transplanted with LSD1‐197‐211 expressed hBTICs also showed longer survival time than mice transplanted with control GFP expressed hBTICs (Figure 6F). Hence, LSD1‐197‐211 inhibits BTICs in vivo as well. However, LSD1‐197‐211 peptide could not inhibit glioma cell lines LN229, T98G and U251 (Figure S5A). This might be due to that these glioma cells do not rely on NR2E1 for cell growth (Figure S6F). Hence, LSD1‐197‐211 peptide should be used together with chemotherapy reagent such as TMZ in brain tumour treatment for sound prognosis.

LSD1 is broadly expressed in most organ tissue with relatively high level in the brain and liver. While NR2E1 is mainly expressed in the brain (Figure S8A). Hence, it is expected that LSD1‐197‐211 peptide which is designed to target NR2E1 and LSD1 interaction would not affect other tissue. To test it, synthesized TAT‐LSD1‐197‐211 peptide and control TAT‐GFP peptide were intravenously injected into nude mouse. TAT peptide was added in front of LSD1‐197‐211 to enable the peptide to penetrate cell membrane. As compared to the control TAT‐GFP peptide, injection of TAT‐LSD1‐197‐211 peptide revealed no significant change in body weight and tissue structure (Figure S8B,C). The level of ALT and AST—the liver damage markers, CR—the kidney damage marker and CKMB—the heart damage marker in mouse blood serum revealed that no damage occurred in the organs (Figure S8D). Hence, LSD1‐197‐211 peptide does not provoke toxic effect on the tested tissues.

## 4 DISCUSSION

High‐grade glioma, including glioblastoma, is the most common primary malignant brain tumour. The general treatment for high‐grade glioma includes surgery, radiotherapy and chemotherapy. However, it is virtually impossible to completely resect these infiltrative tumours and concurrent radiotherapy and chemotherapy do not provide any significant survival benefit for patients. Five‐year survival ratio of patient is still less than 5%. Therefore, novel treatment strategies are desperately needed for this grim disease. BTICs are responsible for the growth and relapse of brain tumour. Therefore, Elimination of them may improve the clinic outcome. Our study revealed that BTICs not only express cancer stem cell markers Nestin and CD133,^32^, ^33^, ^34^, ^35^ they can also differentiate into multiple neural lineages. Importantly, they rely on NR2E1 and LSD1, a transcriptional and epigenetic regulatory mechanism for the cell growth, which provides an interesting target to explore means to inhibit BITCs.

Past clinical research shows that drugs that target epigenetic modifiers yield promising survival benefits in multiple diseases. For example, the use of valproic acid, an HDAC inhibitor, together with radiotherapy, has shown a greater efficacy in GBM patients.^36^, ^37^ In recent years, LSD1 has been deemed as a very promising target, owing to its broad role in cancer, neurodegeneration and viral infection.^38^, ^39^, ^40^, ^41^ However, no therapeutics targeting LSD1 have been developed at present. One of the reasons is that LSD1 is broadly expressed in mammalian tissues, in particular stem cells. Inhibition of LSD1 by AO inhibitors or depletion of LSD1 might therefore cause a significant disturbance of its normal physiological function, leading to unwanted side effects. All reported LSD1 inhibitors that bind to the FAD or AO domains are far from ideal, either because of poor selectivity or their polycationic nature.^12^, ^42^, ^43^ Potential short peptides that could compete with natural histone H3 substrates or bind at sites beyond the active site of the AO domain by an allosteric mechanism to prevent LSD1 from forming complexes or binding to the nucleosomes are being actively explored.^14^, ^44^, ^45^ Nevertheless, targeting the general LSD1 repressive complex, CoREST/LSD1 or LSD1 binding to the histone tail would still disturb the general function of LSD1 in cells. To achieve more effective and specific treatment of high‐grade glioma, targeting the LSD1 involved specific cell proliferation mechanism is more appropriate, with minimum side effects.

Yokoyama et al. have shown that in retinoblastoma cells the DNA binding domain (DBD) of NR2E1 interacts with the AO domain of LSD1, while the ligand‐binding domain (LBD) of NR2E1 interacts with both the SWIRM and AO domains of LSD1.^10^ However, the details of these interactions are not clear. Our study has identified four possible NR2E1‐interacting peptides of LSD1 using HDX‐MS in vitro. HDX‐MS result suggests that amino acid 354–357 of LSD1 contributes to the strongest interaction with NR2E1 in vitro. However, as the protein interaction in vitro is generally affected by the solution cues like pH and the salt concentration, etc., HDX‐MS result just provides possible clues of protein interaction region for further investigation. In other words, the result of deletion mutant study using cell lysate is more reliable than HDX‐MS result to reveal the protein interaction in cells. In addition, prediction of the interaction between LSD1 and NR2E1 LBD using ZDOCK program also shows that the solvent accessible area of LSD1‐354‐377 is likely to be smaller than that of LSD1‐197‐211 (Figure 3D), indicating that LSD1‐354‐377 may contribute to a weaker interaction between LSD1 and NR2E1 than LSD1‐197‐211 (Table S2). This is consistent with the deletion mutant study result. In summary, overexpression of LSD1‐197‐211 efficiently blocks the function of NR2E1 and LSD1 complex and disrupts the demethylation activity of LSD1 at the *Pten* promoter and leads to its upregulation, and therefore, inhibits the proliferation of BTICs (Figure 6G).

However, we should point out that a number of glioblastomas harbour *Pten* mutation. In this case, LSD1‐197‐211 peptide is likely ineffective in these *Pten* mutated glioblastomas. Besides, since the NR2E1 and LSD1 mechanism is also employed by NSCs for self‐renewal, we anticipate that LSD1‐197‐211 may also inhibit NSC proliferation. If this is the case, neurogenesis disturbance that might result from the use of this peptide for brain glioma treatment would be a concern. It is known that neurogenesis is most active in the foetus and reduces with aging. Elderly people, in whom neurogenesis is very low, have the highest probability of developing high‐grade brain glioma. LSD1‐197‐211 may therefore hold great therapeutic potential for patients of this age group. Besides, research has revealed that the regulatory factors of the self‐renewal of NSCs in vitro do not always affect neurogenesis in vivo. For example, mice with mutated inhibitors of DNA binding 1 (Id1), a factor that is required for the self‐renewal of NSCs in vitro, show normal neurogenesis and NSC population in vivo.^3^ On the other side, NR2E1 is seldom detected in non‐neural lineage tissues. Hence, LSD1‐197‐211 is not expected functional in these tissues. Indeed, acute toxicity assays revealed that LSD1‐197‐211 did not provoke any non‐toxic effect in main organ tissues, including Nestin positive tissues like heart and lung. Our study revealed that LSD1‐197‐211 inhibits Nestin and CD133 positive BTSCs. Nestin and CD133 are also expressed in other cancer stem cells, like hepatocellular carcinoma stem cells.^33^, ^34^, ^35^ Whether LSD1‐197‐211 can inhibit those cancer stem cells for cancer treatment is worthy of further exploration.

Overall, our study revealed that LSD1‐197‐211 may serve as a leading peptide for peptide drug development for glioblastoma. Of course, numerous difficulties need to be conquered to bring the discovery to application. Among them, how to deliver the peptide through blood–brain barrier (BBB) to impede into glioblastoma cells is a huge impediment. Due to this common difficulty in the field, we are unable to directly apply the peptide to treat brain tumour at the moment. Even we proved in the study that this peptide can inhibit hBTICs in vivo, it is still a long way to go to apply this peptide for medical application. To date, some preclinical and clinical studies regarding peptide application in brain tumour treatment have been carried out.^46^ Multiple cell‐penetrating peptides have been studied for delivering peptide, nucleic acids or chemicals into brain tumours.^46^ For example, Ueda et al. made a D‐isomer peptide dPasFHV‐p53C′ peptide, which contains cell penetrating peptide CPP, penetration accelerating sequence (PAS) and apoptosis inducer C‐terminus of p53 (p53C′). This peptide could increase the survival rate when administrated to the intracranial GBM mouse model.^47^ The kind of studies inspires us to explore the means of peptide delivery for GBM treatment in future. Besides, we will also try different peptide wrapping materials, such as nanoparticles conjugated with cell penetrating peptides to see whether the peptide delivery efficiency could be increased. To facilitate peptide delivery, we will also explore whether a shorter LSD1‐197‐211 based peptide could be still effective to inhibit BTICs. In summary, further investigation on the peptide delivery, safety and stability optimization will be needed to bring the discovery closer to application.

## AUTHOR CONTRIBUTIONS

Conceptualization: Ping Yuan, Kunchithapadam Swaminathan and Yonggao Mou; methodology: Rong Hu, Umar Farook Shahul Hameed, Philip D. Jeffrey and Jianfeng Pei; formal analysis: Rong Hu, Umar Farook Shahul Hameed, Philip D. Jeffrey and Fangjin Chen; investigation: Rong Hu, Umar Farook Shahul Hameed, Xiang Sun, Balakrishnan Shenbaga Moorthy, Wen Zhang, Li Zhou, Xin Ma and Pankaj K. Giri; resources: Yonggao Mou; writing of the original draft: Ping Yuan; review and editing of the manuscript: Rong Hu, Kunchithapadam Swaminathan and Ping Yuan; supervision: Jianfeng Pei, Kunchithapadam Swaminathan and Ping Yuan; funding acquisition: Yonggao Mou, Kunchithapadam Swaminathan and Ping Yuan.

## CONFLICT OF INTEREST

The authors declare no conflicts of interest

## BIOLOGICAL MATERIAL AVAILABILITY STATEMENT

All biological materials in this study are available from the corresponding author upon reasonable request and in accordance with relevant regulation.

## Supporting information


**FIGURE S1** NR2E1 and LSD1 knockdown by different shRNAs in BTICs

**FIGURE S2** NR2E1 and LSD1 knockdown by RNAi revealed their regulation on PTEN

**FIGURE S3** Inducible lentivirus system of LSD1‐197‐211

**FIGURE S4** Stable expression of LSD1‐197‐211 inhibits the proliferation of BTICs

**FIGURE S5** Alignment of LSD1 SWIRM with previously reported LSD1

**FIGURE S6** LSD1‐197‐211 function in human BTICs

**FIGURE S7** LSD1‐197‐211 function on human BTIC brain tumour formation

**FIGURE S8** LSD1‐197‐211 function in glioma cells and toxicity in mouse

**TABLE S1** Crystallographic data of LSD1 SWIRM domain

**TABLE S2** Complex clusters with buried surface area of NR2E1 by LSD1‐197‐211 and other peptides

## Data Availability

The raw data that supports the findings of this study are available from the corresponding author upon reasonable request.
