# Supplementary figures and images for: A NR2E1‐interacting peptide of LSD1 inhibits the proliferation of brain tumour initiating cells
Source: Cell Prolif. 2022 Nov 2;56(1):e13350. doi: 10.1111/cpr.13350 (PMC9816925; doi:10.1111/cpr.13350)

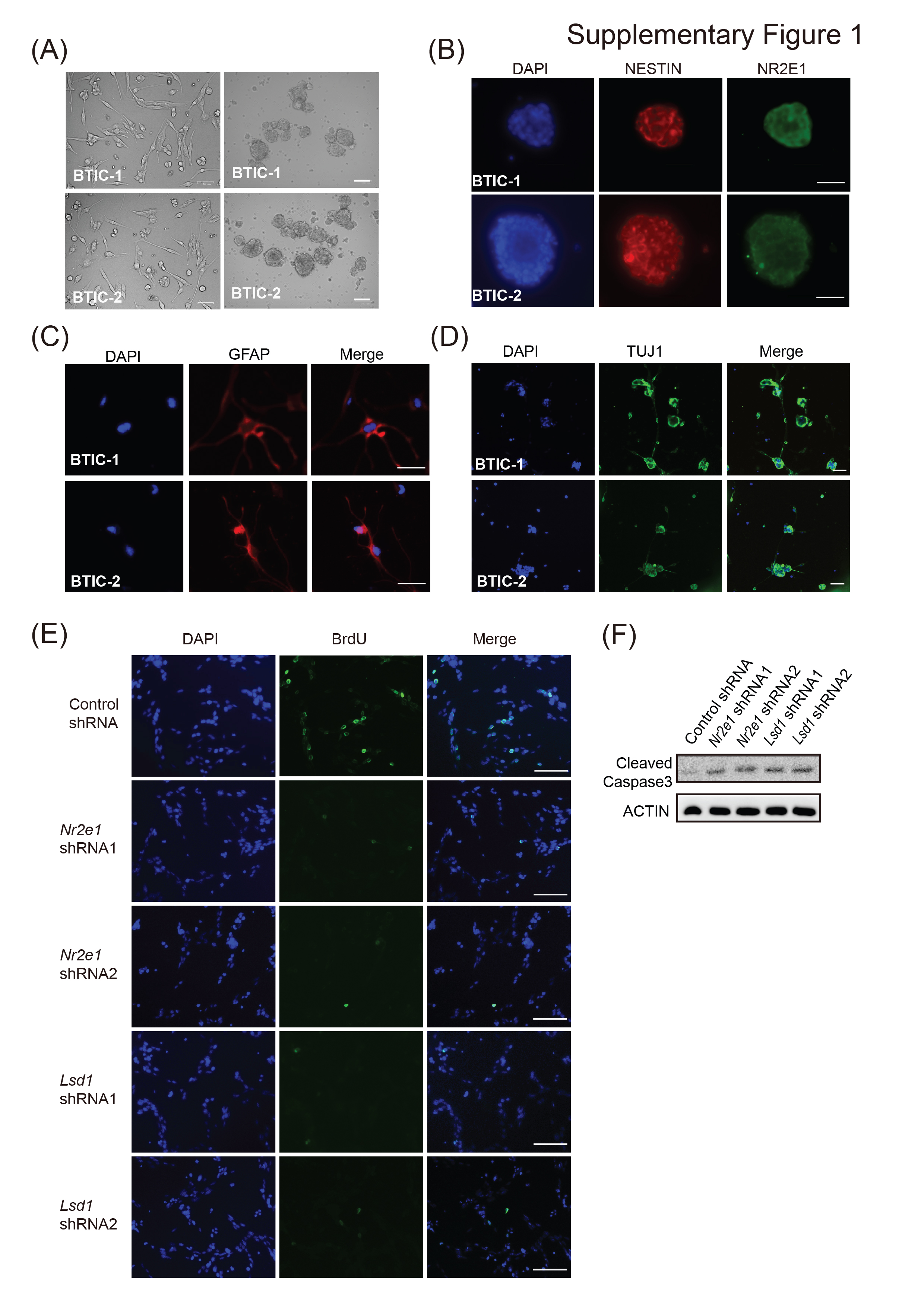

Supplement: Supplementary file 1 — FIGURE S1 NR2E1 and LSD1 knockdown by different shRNAs in BTICs [file CPR-56-e13350-s006.jpg]

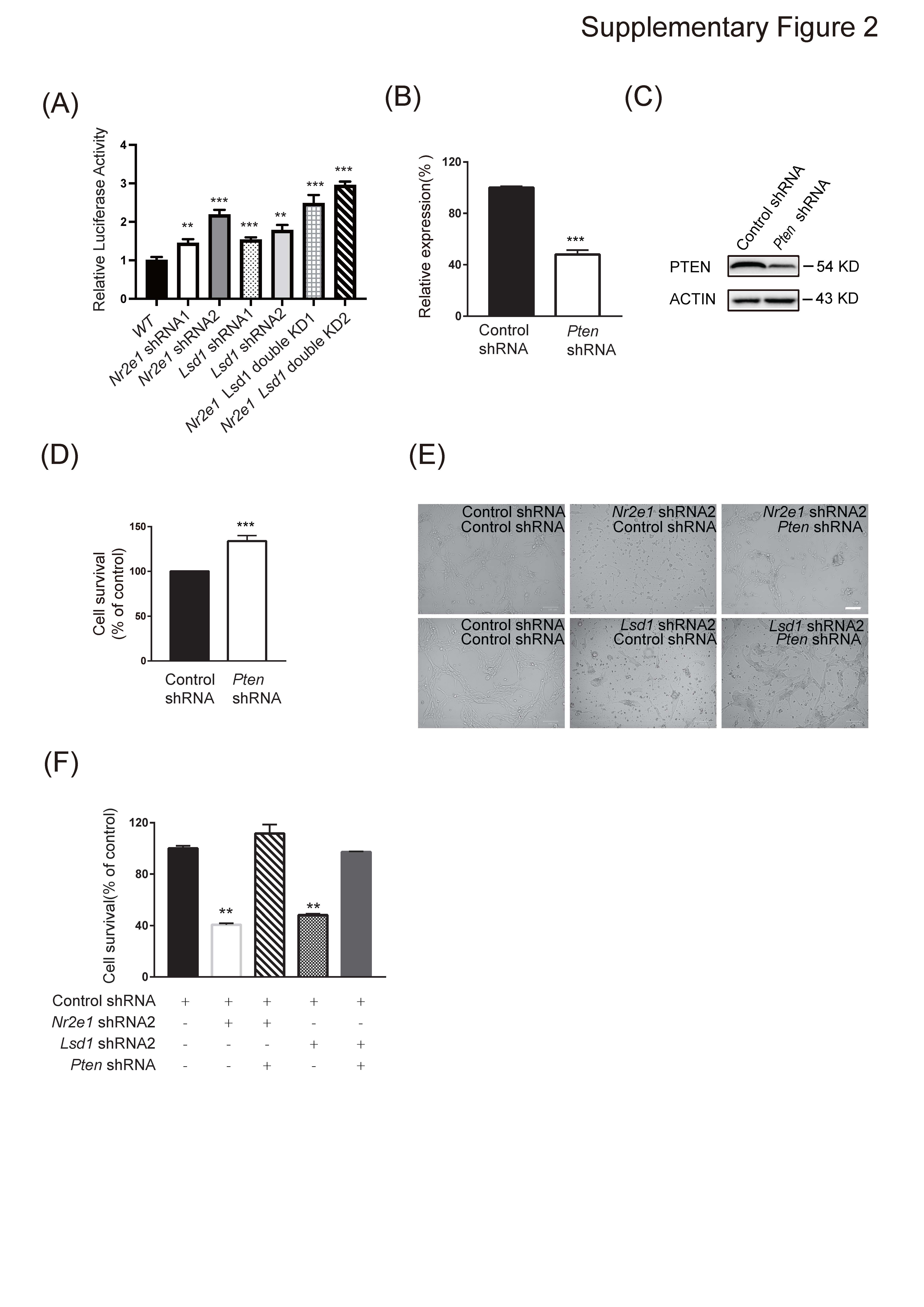

Supplement: Supplementary file 2 — FIGURE S2 NR2E1 and LSD1 knockdown by RNAi revealed their regulation on PTEN [file CPR-56-e13350-s004.jpg]

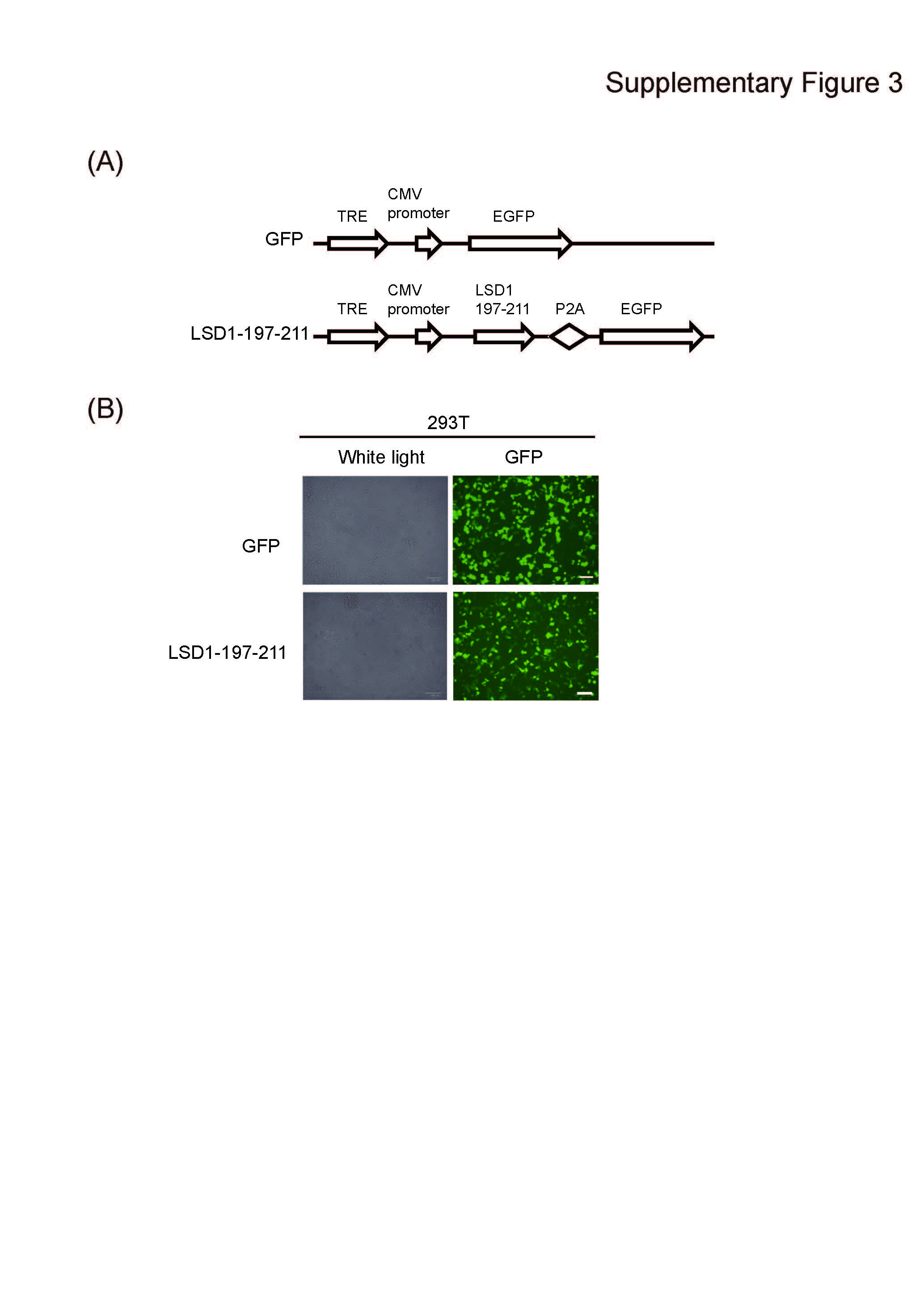

Supplement: Supplementary file 3 — FIGURE S3 Inducible lentivirus system of LSD1‐197‐211 [file CPR-56-e13350-s010.jpg]

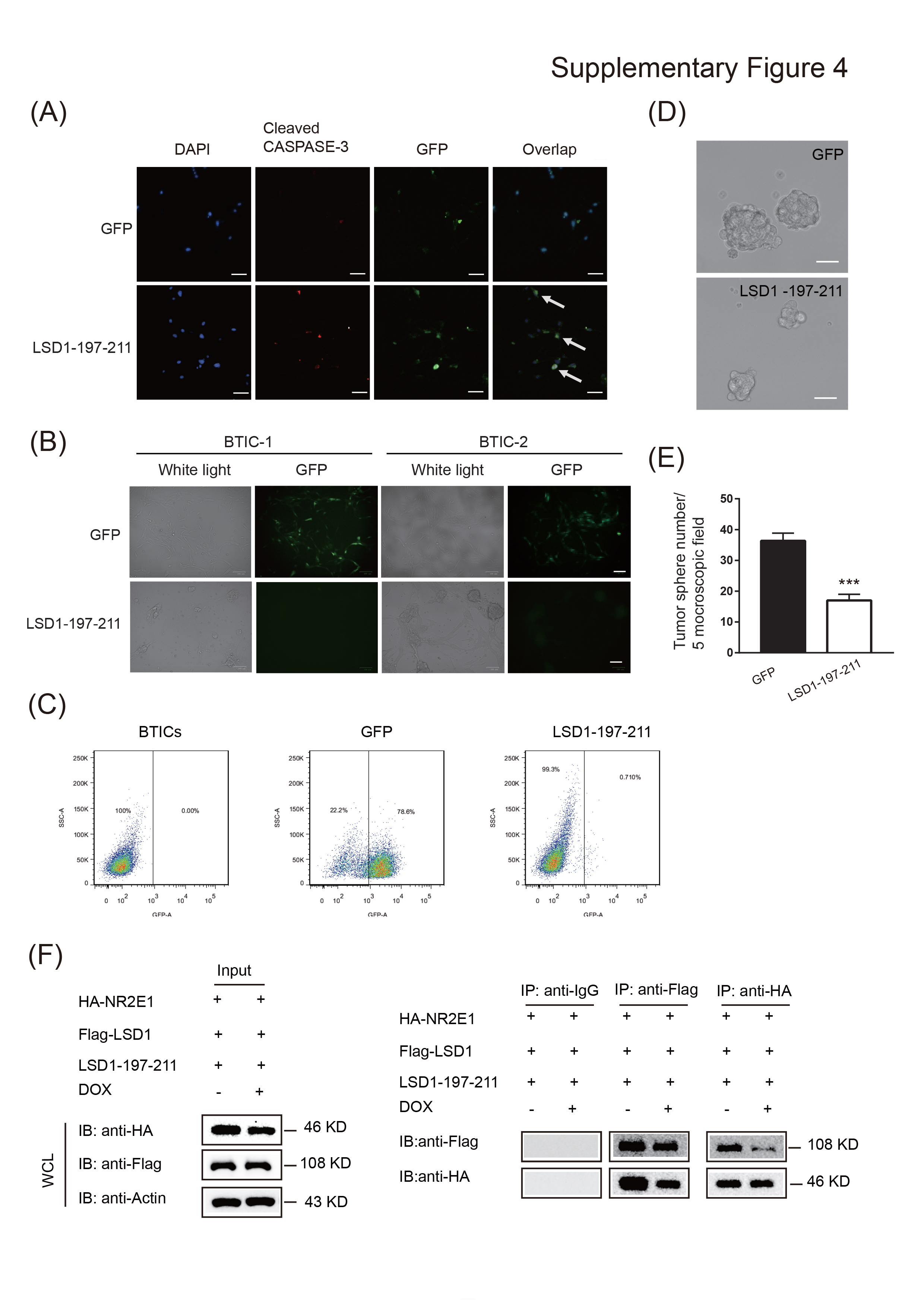

Supplement: Supplementary file 4 — FIGURE S4 Stable expression of LSD1‐197‐211 inhibits the proliferation of BTICs [file CPR-56-e13350-s005.jpg]

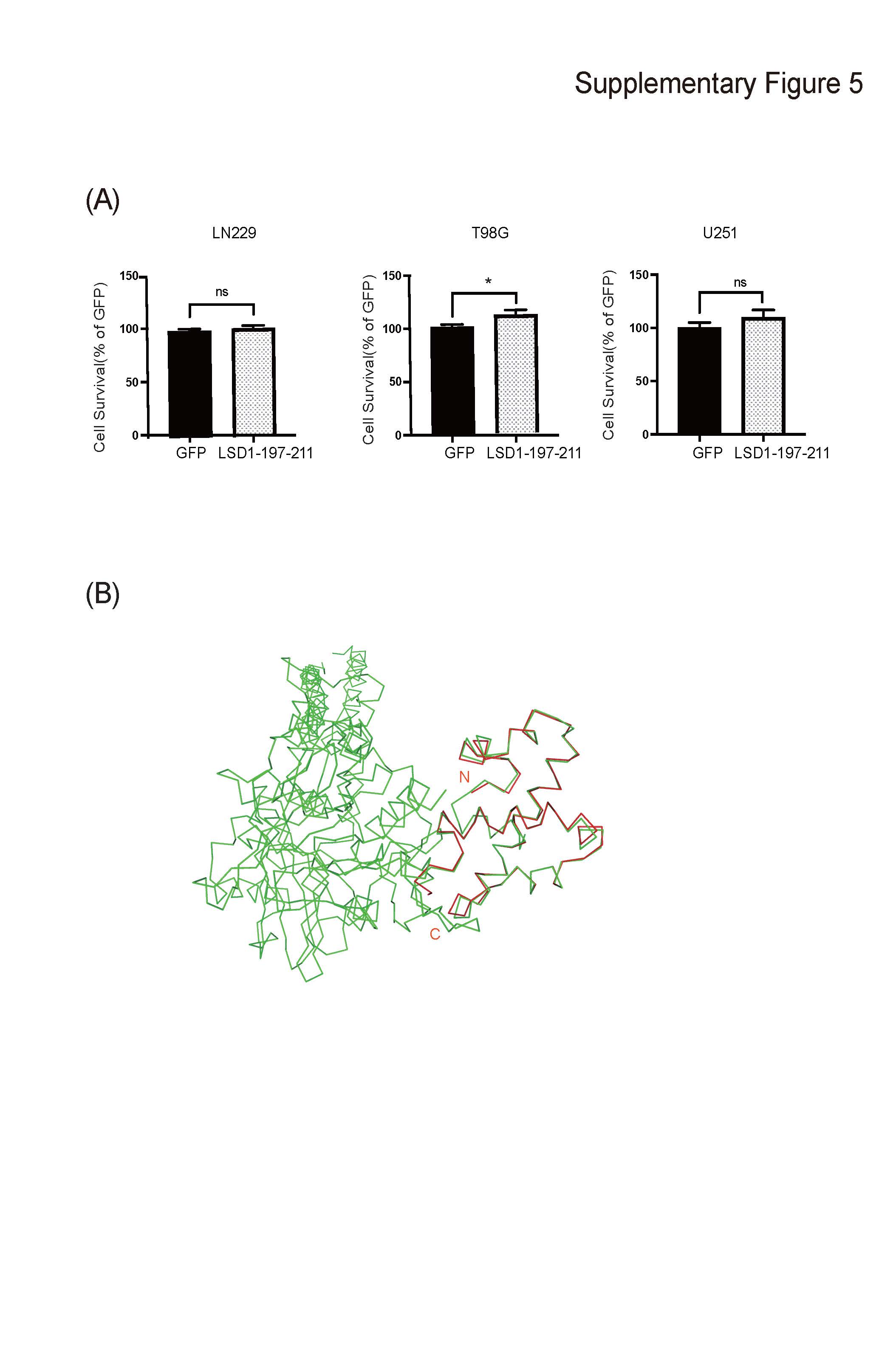

Supplement: Supplementary file 5 — FIGURE S5 Alignment of LSD1 SWIRM with previously reported LSD1 [file CPR-56-e13350-s002.jpg]

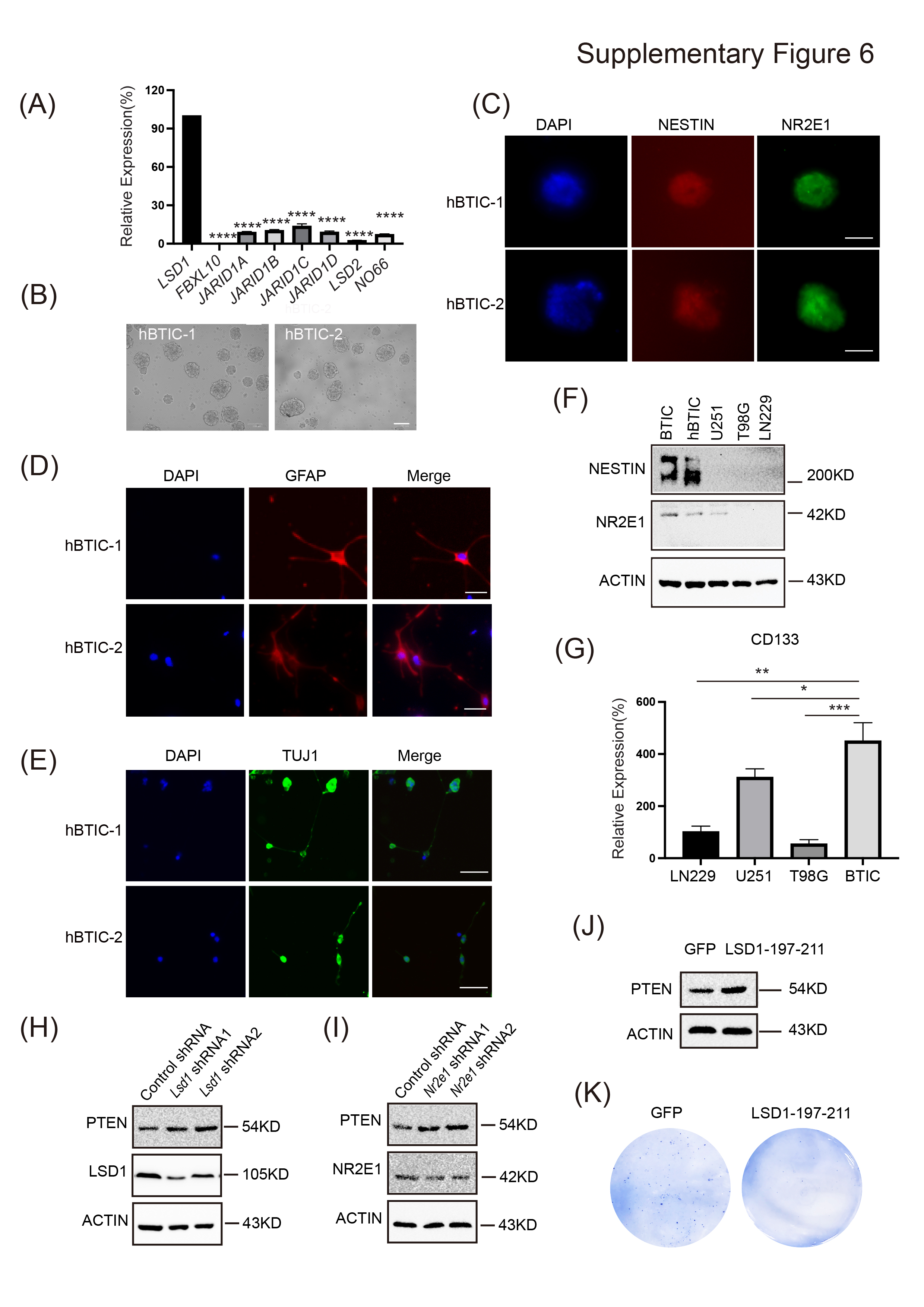

Supplement: Supplementary file 6 — FIGURE S6 LSD1‐197‐211 function in human BTICs [file CPR-56-e13350-s003.jpg]

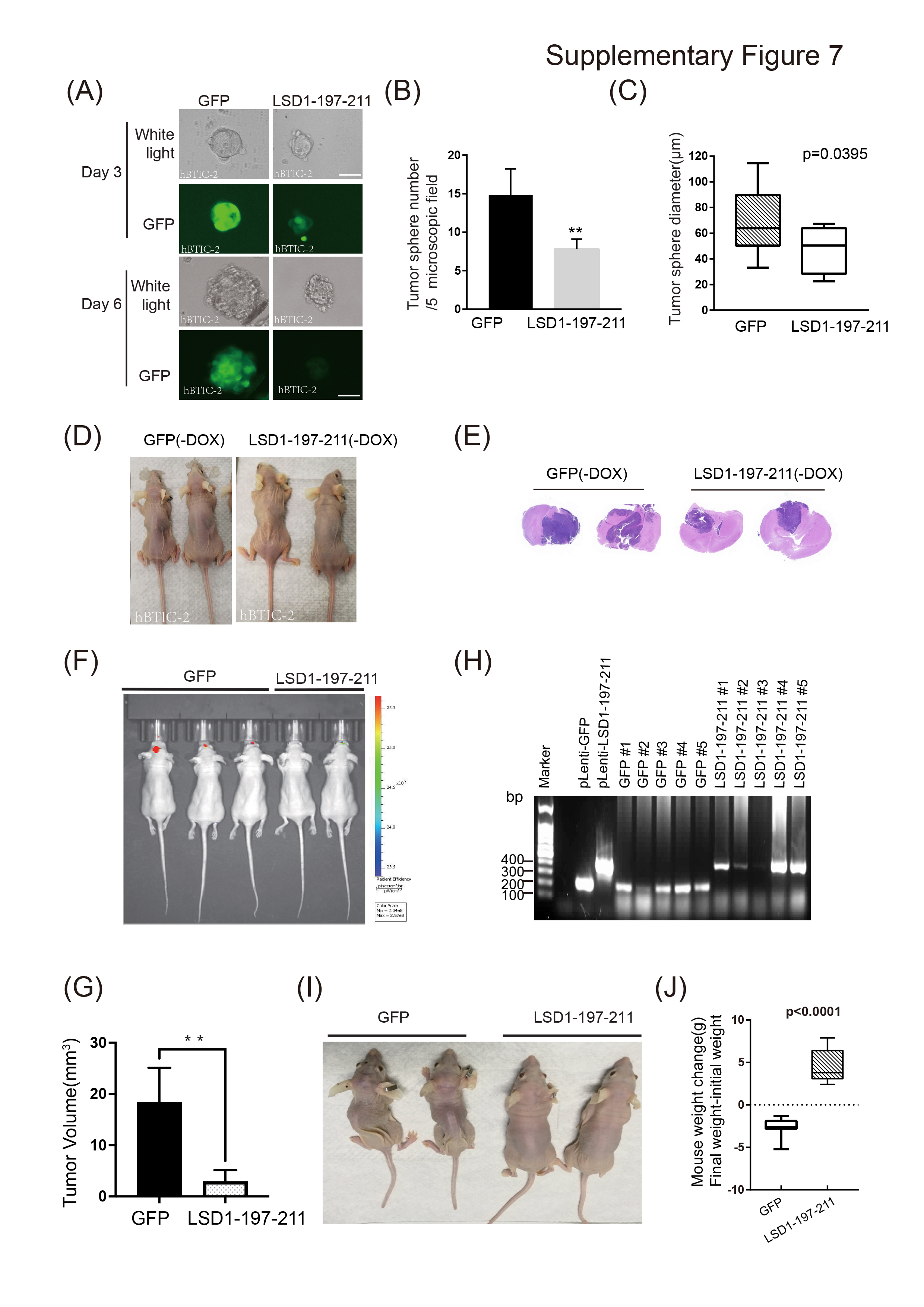

Supplement: Supplementary file 7 — FIGURE S7 LSD1‐197‐211 function on human BTIC brain tumour formation [file CPR-56-e13350-s007.jpg]

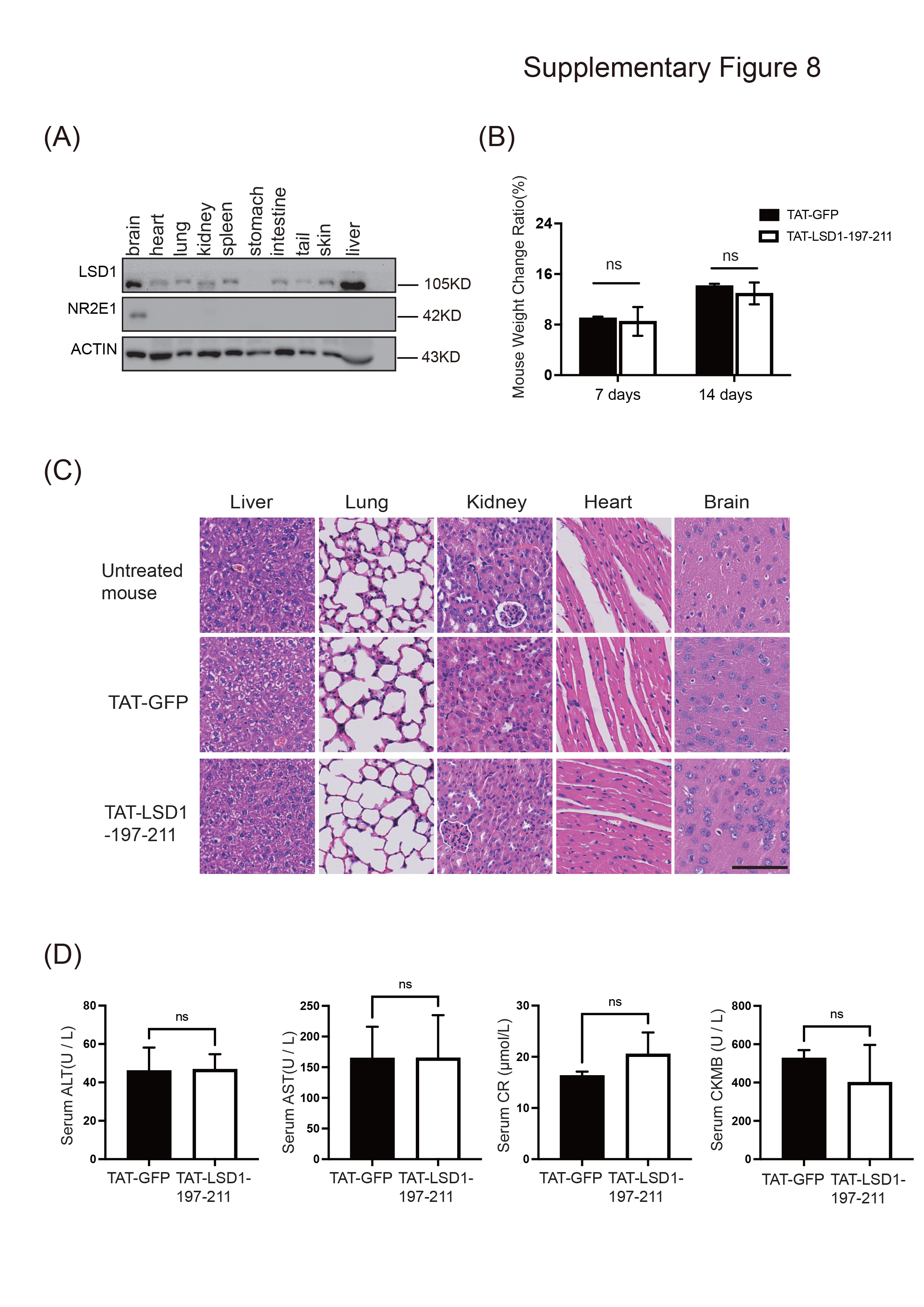

Supplement: Supplementary file 8 — FIGURE S8 LSD1‐197‐211 function in glioma cells and toxicity in mouse [file CPR-56-e13350-s009.jpg]
