# Supplementary material for: A NR2E1‐interacting peptide of LSD1 inhibits the proliferation of brain tumour initiating cells
Source: Cell Prolif. 2022 Nov 2;56(1):e13350. doi: 10.1111/cpr.13350 (PMC9816925; doi:10.1111/cpr.13350)
Supplement: Supplementary file 9 — TABLE S1 Crystallographic data of LSD1 SWIRM domain [file CPR-56-e13350-s001.docx]

Supplementary Table 1. Crystallographic data of LSD1 SWIRM domain

**Crystallographic data**

Native crystal Native crystal

Space group P2_1_2_1_2 P2_1_2_1_2_1_

Unit cell dimension (Å) a=57.17, b=49.97, c=48.85 a=48.86, b=52.37, c=60.86

Selenomethionine crystal I222

a=41.50, b=68.03, c=68.23

| ***Data collection*** |  | | | | | | |
| --- | --- | --- | --- | --- | --- | --- | --- |
| Data sets | Native 1 | Native 2 | Peak | Inflection |  | Remote 1 | Remote 2 |
| Wavelength (Å) | 0.9777 | 0.9777 | 0.9790 | 0.9793 |  | 0.9649 | 0.9800 |
| Resolution (Å) | 100-1.40 | 100-1.50 | 100-2.00 | 100-1.90 |  | 100-1.90 | 100-1.90 |
| Outer shell (Å) | 1.45-1.40 | 1.55-1.50 | 2.07-2.00 | 1.97-1.90 |  | 1.97-1.90 | 1.97-1.90 |
| Unique reflections | 28163 | 21576 | 13974 | 14524 |  | 14505 | 7876 |
| Redundancy | 3.8 | 3.2 | 3.1 | 3.2 |  | 3.2 | 5.1 |
| Completeness, % | 99.8 (100.0) | 96.0 (84.0) | 96.3 | 97.4 |  | 97.1 | 98.3 |
| Rsym  Mean figure of merit | 0.040 (0.292) | 0.045 (0.316) | 0.042 | 0.042 | 0.58 | 0.042 | 0.039 |
| ***Refinement parameters*** |  |  |  | | | | |
| Resolution (Å) | 50-1.4 | 40-1.55 |  |  |  |  |  |
| Reflection, work/free | 25553/1358 | 19835/1082 |  |  |  |  |  |
| completeness, % | 95.12% | 90.30% |  |  |  |  |  |
| No. of atoms | 1646 | 1558 |  |  |  |  |  |
| No. of waters | 203 | 154 |  |  |  |  |  |
| Rwork | 0.159 | 0.163 |  |  |  |  |  |
| Rfree | 0.194 | 0.197 |  |  |  |  |  |
| rmsd bond length (Å) | 0.012 | 0.013 |  |  |  |  |  |
| rmsd bond angles (°) | 1.47 | 1.59 |  |  |  |  |  |
| Average B-factor (Å^2^) |  |  |  |  |  |  |  |
| Protein | 20.51 | 24.26 |  |  |  |  |  |
| Water | 32.89 | 34.14 |  |  |  |  |  |
| ***Ramachandran plot*** |  |  |  |  |  |  |  |
| Within favored, % | 98.50% | 99.40% |  |  |  |  |  |
| Within allowed, % | 100% | 100% |  |  |  |  |  |
| Outliers, % | 0 | 0 |  |  |  |  |  |
