# Supplementary material for: A NR2E1‐interacting peptide of LSD1 inhibits the proliferation of brain tumour initiating cells
Source: Cell Prolif. 2022 Nov 2;56(1):e13350. doi: 10.1111/cpr.13350 (PMC9816925; doi:10.1111/cpr.13350)
Supplement: Supplementary file 10 — TABLE S2 Complex clusters with buried surface area of NR2E1 by LSD1‐197‐211 and other peptides [file CPR-56-e13350-s008.docx]

Supplementary Table 2. Complex clusters with buried surface area of NR2E1 by LSD1-197-211 and other peptides

| Docking Clusters | Buried surface area (Å^2^) of NR2E1 by | | | |
| --- | --- | --- | --- | --- |
|  | LSD1-197 -211 | LSD1-354 -377 | LSD1- 481 -501 | LSD1-537 -546 |
| Cluster_107 (8)^*^ | 493.3 | 67.6 | 0 | 0 |
| Cluster_256 (4) | 522.6 | 305.2 | 0 | 0 |
| Cluster_25 (5) | 221.5 | 174.2 | 0 | 0 |
| Cluster_278 (2) | 258.5 | 189.1 | 0 | 0 |
| Cluster_350 (7) | 183.2 | 261.7 | 0 | 0.1 |
| Cluster_38 (9) | 503.6 | 141.3 | 0 | 0 |
| Cluster_45 (8) | 137.4 | 77.6 | 0 | 0 |
| Cluster_465 (1) | 531.2 | 467.3 | 0 | 0 |
| Cluster_536 (5) | 238.4 | 179.2 | 0 | 0 |
| Cluster_577 (1) | 55.4 | 172.7 | 0 | 0 |
| Cluster_592 (3) | 353 | 223.5 | 0 | 0 |
| Cluster_63 (3) | 453 | 313.6 | 0 | 0 |
| Cluster_82 (16) | 154.8 | 101.4 | 0 | 0 |

* Numbers in the brackets are docking complex numbers in the same cluster.
